# The functional epigenetic landscape of aberrant gene expression in molecular subgroups of newly diagnosed multiple myeloma

**DOI:** 10.1186/s13045-020-00933-y

**Published:** 2020-08-06

**Authors:** Samrat Roy Choudhury, Cody Ashby, Ruslana Tytarenko, Michael Bauer, Yan Wang, Shayu Deshpande, Judith Den, Carolina Schinke, Maurizio Zangari, Sharmilan Thanendrarajan, Faith E. Davies, Frits van Rhee, Gareth J. Morgan, Brian A. Walker

**Affiliations:** 1 Myeloma Center, University of Arkansas for Medical Sciences, Little Rock, AR 72205 USA; 2 Department of Medicine, NYU Langone Health, New York, NY 10016 USA; 3 Division of Hematology Oncology, Melvin and Bren Simon Comprehensive Cancer Center, Indiana University, Indianapolis, IN 46202 USA

**Keywords:** myeloma, DNA methylation, gene regulation, epigenetics

## Abstract

**Background:**

Multiple Myeloma (MM) is a hematological malignancy with genomic heterogeneity and poor survival outcome. Apart from the central role of genetic lesions, epigenetic anomalies have been identified as drivers in the development of the disease.

**Methods:**

Alterations in the DNA methylome were mapped in 52 newly diagnosed MM (NDMM) patients of six molecular subgroups and matched with loci-specific chromatin marks to define their impact on gene expression. Differential DNA methylation analysis was performed using DMAP with a ≥10% increase (hypermethylation) or decrease (hypomethylation) in NDMM subgroups, compared to control samples, considered significant for all the subsequent analyses with p<0.05 after adjusting for a false discovery rate.

**Results:**

We identified differentially methylated regions (DMRs) within the etiological cytogenetic subgroups of myeloma, compared to control plasma cells. Using gene expression data we identified genes that are dysregulated and correlate with DNA methylation levels, indicating a role for DNA methylation in their transcriptional control. We demonstrated that 70% of DMRs in the MM epigenome were hypomethylated and overlapped with repressive H3K27me3. In contrast, differentially expressed genes containing hypermethylated DMRs within the gene body or hypomethylated DMRs at the promoters overlapped with H3K4me1, H3K4me3, or H3K36me3 marks. Additionally, enrichment of BRD4 or MED1 at the H3K27ac enriched DMRs functioned as super-enhancers (SE), controlling the overexpression of genes or gene-cassettes.

**Conclusions:**

Therefore, this study presents the underlying epigenetic regulatory networks of gene expression dysregulation in NDMM patients and identifies potential targets for future therapies.

## Introduction

Multiple Myeloma (MM) is characterized by an abnormal clonal plasma cell infiltration in the bone marrow, which may lead to the development of lytic bone lesions and myelosuppression [1, 2]. The etiological genetic features of MM include translocations between the *IgH* locus and a number of oncogenes, including *MMSET*/*FGFR3* (4p16), *CCND1* (11q13), *MAF* (16q23), *MAFB* (20q12), or aneuploidy demonstrated in patients with hyperdiploid genomes [3–5]. In addition to etiological events, secondary acquired genetic abnormalities, including recurrent mutations, have been reported. These acquired genetic abnormalities deregulate key oncogenes and tumor suppressor genes in MM [6].

Few studies in myeloma have attempted to clarify the epigenetic drivers and their impact on the underlying disease, with the majority having focused on global alterations in DNA methylation, histone modifications, and noncoding miRNAs [7–11]. Individual epigenetic marks have been investigated through the use of low-throughput techniques, such as methylation specific PCR, pyrosequencing, and semi-high output 450K methylation arrays [8, 9, 12].

Regarding DNA methylation, we and others have shown that there is a significant change in DNA methylation levels at the transition from monoclonal gammopathy of undetermined significance (MGUS) to MM, resulting in genome-wide hypomethylation while specific genes are hypermethylated [8, 11]. There is also a clear difference in the DNA methylation levels in the t(4;14) MM subgroup compared to other subgroups, and this is thought to be due to over-expression of the histone methyltransferase MMSET in this group. DNA methylation has also been used to identify genes of prognostic interest, highlighting the importance of this biological process [9]. However, the possible internal cross-talk between epigenetic regulators at the DNA and histone levels and their combinatorial effects on gene expression patterns in different MM molecular subgroups has not been addressed.

To address this deficiency, we have optimized the use of enhanced reduced representation bisulfite sequencing (eRRBS), complemented with 850K methylation array (Illumina), in newly diagnosed MM (NDMM) patients of six molecular subgroups to determine the alterations in DNA methylome per subgroup in order to compare to healthy donors. Enrichment of promoter and gene body-associated CpG sites allows robust correlation between DNA methylation at differentially methylated regions (DMRs) and expression of the closest gene. Additionally, we show that these DMRs co-localize with other epigenetic factors, including histone marks and SE protein signatures, to impact gene expression dysfunction in MM.

## Methods

### Patients and sample preparation

Fifty two NDMM patients were consented with IRB approval for bone marrow aspirates for CD138+ cell selection (RoboSep, StemCell Technologies, Germany) to enrich for tumor cells at least >90%. Patients represented the major translocation and hyperdiploidy subgroups and were compared to CD138+ PCs isolated from bone-marrow random aspirates of four age-matched healthy donors. These patients were well-characterized in terms of diagnostic variables, demographic, and clinicopathological parameters (**Supplementary Table****1**). DNA and RNA were extracted using AllPrep DNA/RNA mini kit (Qiagen, Hilden, Germany), RNeasy RNA extraction kit (Qiagen), or Puregene DNA extraction kit (Qiagen). Bisulfite conversion of DNA was carried out using EZ-DNA methylation kit (Zymo Research, CA, USA).

### eRRBS sample processing, library preparation, and sequencing

The eRRBS protocol was optimized with 100 ng of genomic DNA. Briefly, DNA samples were digested overnight with MspI followed by end-repair and A-tailing, methylated adapter ligation, uracil removal treatment, magnetic bead-based size selection, bisulfite conversion, and PCR enrichment [13]. The size and concentration of library fractions were determined prior to sequencing. Samples were multiplexed and sequenced using 75-bp single end reads.

### Interpretation of eRRBS data

Quality control of the sequencing reads and methylation base calling were performed using bcl2fastq2 (Illumina) and TrimGalore (v 0.4.4) software, respectively. We obtained an average of 2.198x10^7^ total aligned reads per sample and measured the methylation levels of an average of 21 million methylated CpG sites per sample from the eRRBS data (**Supplementary Table****1**). Sequencing data were aligned to whole genome version hg38/GRCh38 using the Bismark alignment software (v 13.0) (Babraham Bioinformatics, UK). Differential methylation analysis was performed using DMAP (v 1.42) [14] and cytosines with fewer than 10 reads in any sample were discarded from subsequent analyses. Bismark quality control report on the eRRBS data are listed in **Supplementary Table **2. 

DMRs containing at least 2 CpG sites were considered for subsequent analyses, provided the methylation percentage of both the control and NDMM groups were not >80%, <20%, or between 40% and 60%. DMRs of ≥10% (a false discovery rate [FDR]-adjusted p-value <0.05) increase (hypermethylation) or decrease (hypomethylation) in NDMM subgroups, compared to control samples, were considered significant for all the subsequent analyses. DMRs were annotated by identifying the closest TSS according to RefSeq. Regions that were 5 kb upstream and 200 bp downstream from the TSS were marked as promoter regions, while the region immediately downstream of the defined promoters to the 3’ ends were marked as gene body. Relative distance percentage (D) of a DMR from the nearest TSS site was calculated using the following formula:

D= (Absolute distance from the end base of DMR-TSS start base/Gene length) X 100

Genes with the combination of both hyper- and hypomethylated DMRs at promoter or body are excluded from the present study. An unsupervised hierarchical clustering was performed using the top 5% most variable CpG sites using hclust method in R.

### MethylationEPIC 850k bead array

Methylation array was performed in 48 patients, including 34 of the 52 NDMM patients of the eRRBS dataset in addition to 14 NDMM samples. 500 ng of genomic DNA was used as input**.** Genomic DNA were bisulfite converted and processed on Infinium HumanMethylationEPIC BeadChip arrays (Illumina Inc., CA, USA) per manufacturer’s protocol. Microarray raw IDAT and annotation files (Infinium MethylationEPIC v1.0_B4 Manifest File) for the EPIC assay were loaded into GenomeStudio software (v 1.9.0, Illumina) for differential methylation analysis. Difference in the average methylation proportion (β value) at a CpG site between control and NDMM patients >0.1 were considered significant at a diffscore >+/-13 (equivalent to adjusted p-value 0.05). All downstream analyses were conducted by converting the hg19/GRCh37 coordinates of EPIC probe-sets (default in GenomeStudio) to hg38/GRCh38 using the UCSC lift-over tool to match and validate the eRRBS data, wherever possible.

### Gene expression profiling

Gene expression profiling (GEP) using Affymetrix U133 Plus 2.0 arrays was performed. CEL files were processed using the Transcriptome Analysis Console (v 4.0, Thermo Fisher, CA, USA), where raw intensity values were MAS5 normalized and converted to log_2_ scale. Average GEP value over 2-fold more or less in NDMM patients compared to controls were defined as overexpressed and under-expressed genes respectively.

### RNA sequencing

RNA sequencing was performed on 45 out of the 52 NDMM patients (as specified in **Supplementary Table 1**). RNA-seq was performed using 100 ng total RNA with genomic DNA removal using the TURBO DNA-free kit (Ambion). RNA was prepared using the TruSeq stranded total RNA Ribo-zero gold kit (Illumina) and libraries were sequenced using 75 bp paired end reads on a NextSeq500 (Illumina). RNA-seq data was analyzed using the transcript aligner STAR (v2.5.1b) [15] and transcript level data were generated by Salmon (v0.7.2) [16]. RNA-sequencing and microarray data were compared for key genes per NDMM subgroup (**Supplementary Figure 11**).

### Intersect analysis of methylation and expression

A Venn intersection analysis was carried out using Venny (v 2.0) to identify the hypermethylated under-expressed and hypomethylated overexpressed genes at the promoter. Additionally, the methylation and expression correlations were determined on hypomethylated-under-expressed and hypermethylated-overexpressed gene clusters at the gene bodies.

### Gene ontology and protein interaction prediction

Gene clusters per MM subgroup and their possible involvement in biological processes and molecular functions were determined using the GO enrichment analysis platform (http://geneontology.org/page/go-enrichment-analysis) at FDR <0.05. Possible functional protein interactions and involvement in KEGG annotated pathways of the gene clusters were further analyzed with STRING consortium (https://string-db.org/cgi/network.pl) tools (v 10.5).

### Histone modifications (ChIP-seq) analysis

Chromatin immunoprecipitation and fixation were carried out per manufacturer’s protocol (Active Motif Inc.) on mycoplasma free and STR (short tandem repeat) checked KMS11 and MM1.S cells. Antibodies were used to determine genome-wide four activating histone marks, namely H3K4me3, H3K27ac, H3K4me1, and H3K36me3, and two inactivating histone marks, namely H3K9me3 and H3K27me3 and data are available under accession number GSE151556. Purchase of ChIP antibodies, experiments, and analyses of the data were carried out in association with Active Motif Inc. (CA, USA). The histone marks for the U266 cell line were obtained from the Blueprint epigenome consortium (http://www.blueprint-epigenome.eu/). MACS2 was used for peak calling to determine the enrichment of histone marks both at the short and larger overlapping DMRs [17]. Delineation of significantly ChIP-enriched regions were made in the form of SICER BED files and [18] were uploaded in UCSC custom track to align histone marks to the DNA and SE-CTCF modifications.

### Annotations for CTCF, super-enhancer, and enhancer sites

Possible overlap of CTCF sites with the DMRs were determined by using ChIP-seq data for CTCF in the delta47 myeloma cell line [19]. Possible overlaps of SE with DMRs were determined by using ChIP-seq data from Lovén et al. 2013, from BRD4 binding in the MM1.S [20] cell line. The clustering of the SE/CTCF-DMRs were performed by determining the average linkage of DMR-methylation level using Euclidian distance method.

### Statistical analysis

A two-way ANOVA was used to determine the significance in differential methylation analysis. A non-parametric two tailed Mann-Whitney U test was performed for rest of the analysis and statistical analyses were determined at a p value < 0.001 or < 0.05 (as indicated).

## Results

### Differential DNA Methylation Patterns Define NDMM Molecular Subgroups

Using eRRBS, an unbiased genome-wide DNA methylation analysis of sorted CD138+ bone marrow (>98% enriched for tumor) plasma cells (PCs) from 52 NDMM patients was performed (**Supplementary Table 1**). Our sample set contained four of the major *IgH* etiologic translocation MM subgroups, including t(4;14), t(11;14), t(14;16), and t(14;20) and two hyperdiploid (HRD) MM subgroups, defined by overexpression of *CCND1* (HRD-D1) or *CCND2* (HRD-D2) (**Supplementary Table****2**).

**Fig. 1:**
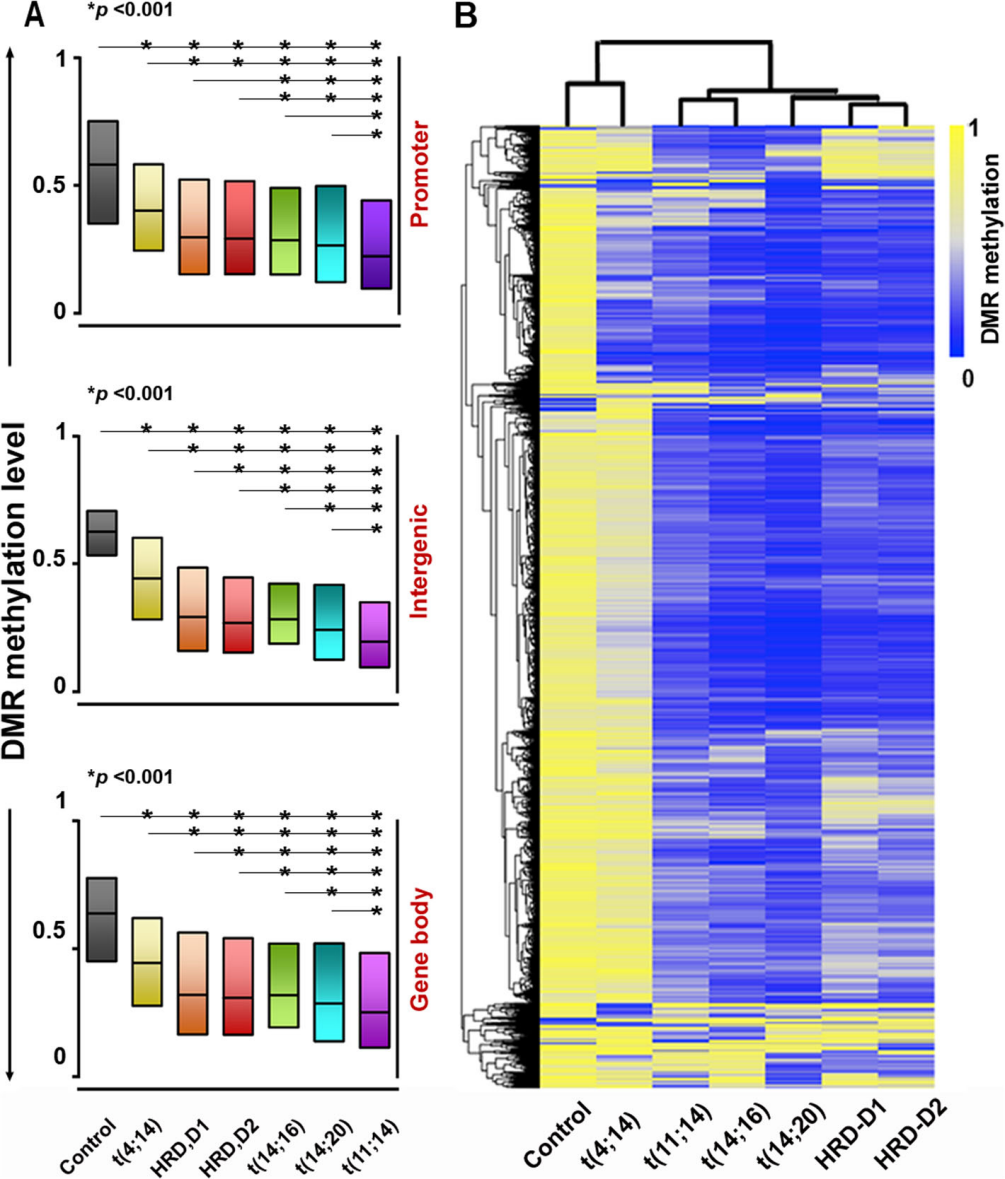
DNA methylation differs by myeloma subgroup. (**a**) The median DNA methylation level of the top 5% differentially methylated regions (DMRs) of MM subgroups, compared to control (p<0.001), depicted as boxplots with interquartile range between 25% and 75% across the promoters (n=1,282), gene body (n=6,594) and intergenic regions (n=5,050). (**b**) Unsupervised hierarchical clustering with the top 5% most variable DMRs (n=12,931) showed distinct methylation pattern among the NDMM subgroups.

Principle-component analysis (PCA) of CpG methylation at DMRs showed a distinct segregation pattern of t(4;14) samples from the control PCs and remaining subgroups (**Supplementary Figure****1**). The top 5% most variable DMRs (n=12,926 unique regions) among the MM subgroups were used to determine the level of methylation across the corresponding gene bodies, promoters, or intergenic regions (IGR) (**Supplementary Table 3**). The majority of these most variable DMRs were observed within the body (51%), followed by IGRs (39%), and promoters (10%) (**Fig.****1****a,****Supplementary Figure 2A**) with all regions and subgroups showing significant hypomethylation compared to the control. An unsupervised hierarchical clustering of DMRs (*p*<0.001) across the MM subgroups demonstrated relative hypermethylation (19.6%) in the t(4;14) subgroup (**Fig.****1****b**). In contrast, the average number of hypermethylated DMRs were as low as 5.6% in HRD, 4.3% in maf subgroups, and 3.4% in the t(11;14) subgroup. Based on the similarities in DMRs, we also observed close neighboring clusters between the t(14;16) and t(14;20), or between the HRD-D1 and HRD-D2 subgroups. In contrast, the t(4;14) subgroup was distant from all other subgroups, presumably due to the over-expression of MMSET resulting in changes in histone and DNA methylation (**Supplementary Figure 2****B**).

The data obtained from eRRBS were complemented with 850K methylation microarray data to demonstrate differential methylation within and beyond the DMRs. We again used the top 5% most variable differentially methylated cytosines (DMCs) and clustered them to determine subgroup methylation disparities. We observed correlations between the methylation pattern of the most variable DMRs and DMCs across the subgroups, r^2^=0.91. Genomic distribution of the DMCs among the subgroups are tabulated in **Supplementary Table****4**. Based on the PCA and methylation pattern at DMCs, we found similar neighboring DMC-clusters between the t(14;16) and t(14;20), or HRD-D1 and HRD-D2 subgroups, **Supplementary Figures****3 and 4**. Moreover, the t(4;14) was again most distantly separated from the other MM subgroups.

### The Expression Pattern Typical of Molecular Subgroups Are Associated with DNA Methylation

We analyzed gene expression profiles in the same 52 NDMM patients to understand the subgroup-specific differences in transcriptome profile and their possible correlation with the altered methylation (**Supplementary Figure****5****A-F**). Based on clustering analysis of the top 5% most variably expressed genes the t(14;16) and t(14;20) subgroups clustered together, as did the HRD-D1 and HRD-D2 subgroups, while the t(4;14) and t(11;14) subgroups remained isolated from all other subgroups (**Supplementary Figure****5****G**)**,** mirroring the DNA methylation clusters**.**

The hierarchical clustering of DMR/DMC data therefore resulted in a very similar clustering of the expression data, suggesting expression and methylation patterns in NDMM subgroups may be interconnected. We therefore carried out an unbiased intersection analysis containing median methylation level of DMRs versus nearest gene expression per subgroup. In consensus with the literature, we focused on the promoters, where DMRs are inversely correlated with expression, and at the gene body, where DMR-methylation is positively correlated with corresponding gene-expression [21, 22], defining them as genes whose expression correlates with DNA methylation (GEMs). Differential gene-expression of ≥2-fold between NDMM patients and control were matched to the DMRs or the corresponding genes, where differential methylation between NDMM patients and control were ≥10%. We observed that of the genes differentially expressed in each subgroup, GEMs accounted for 36.1% in t(4;14), 39.7% in t(11;14), 42.4% in t(14;16), 47.1% in t(14;20), 45.5% in HRD-D1, and 44.2% in the HRD-D2 subgroup. It was also observed that number of over-expressed GEMs per subgroup were almost two-times (**Supplementary Table****5**) more than the under-expressed ones.

Furthermore, we compared the DMR-density distribution at the promoter and body of the GEMs across the subgroups to have a better understanding of the regional effect of DNA methylation on gene expression. Based on the significance of probability (Pr>F), we showed that the hypomethylated but not the hypermethylated DMRs had a significantly different distribution (**Supplementary Figure****6**) at both the promoter and gene body across the subgroups, and that this difference is mostly due to the t(4;14) subgroup. It was also observed that the t(4;14) GEMs had the lowest number of DMRs, while the t(11;14) and t(14;20) GEMs had the highest number of DMRs, compared to the control. Overall, on average we found >42% of differentially expressed genes in MM are controlled by DNA methylation. We also observed that DNA methylation at the DMRs or DMCs at the gene body were directly proportional to gene expression levels, with a correlation of at least r^2^=0.58 across the subgroups.

### Histone Modifications Are Tightly Linked to the Overlapping DMRs and DMCs

We also assessed the global overlap in activating (H3K4me1, H3K4me3, H3K27ac, and H3K36me3) or repressive (H3K9me3 and H3K27me3) histone marks with DMRs for a better understanding of the underlying epigenetic networks of gene expression in NDMM [23, 24]. We analyzed the DMR-histone overlap (>50 bp) in the t(4;14) MM subgroup using data from KMS11 cell lines, in the t(11;14) with U266 cells and in the t(14;16) with MM1.S cells.

We found H3K27me3 as the most abundant mark on hypomethylated DMRs at both the promoter and gene body in t(4;14), t(11;14) and t(14;16) subgroups (**Supplementary Table****19**). We also observed enrichment of H3K4me3 next to H3K27me3 at the hypomethylated DMRs in promoters.

In contrast, hypermethylated DMRs were predominantly enriched with H3K4me1, H3K4me3, or H3K36me3 at both the promoters and bodies in the representative cell lines. Most interestingly, these gene-body DMRs were primarily correlated to overexpression of the related GEMs (**Supplementary Fig****7****A-C**). This finding supports the hypothesis that similar to other cancers, DNA methylation within the gene body may also play an important role in determining expression in MM [25, 26]. A relatively low amount of H3K27ac was also observed at the promoters or gene-bodies but that relate to at least 14% overexpression in KMS11 t(4;14), 12% in U266 t(11;14), and 15% in MM1.S t(14;16) subgroups. A low fraction of H3K9me3 was also noticed, which did not necessarily correlate to any significant number of gene suppression.

Finally, a bimodal distribution in DMR-histone overlaps were observed at the IGRs in all the cell types. For instance, 80% hypomethylated DMRs in KMS11, 54% in U266, and 67% in MM1.S cells were overlapped to H3K27me3. In contrast, enrichment of H3K4me3 to hypermethylated DMRs in the cell lines suggest the existence of potential distal cis-regulatory control of gene-expression at the IGRs in MM [27]. In summary, DNA methylation and histones in NDMM are tightly linked to each other and involved in diverse array of epigenetic relationships impacting gene expression.

### Epigenetic Transcriptional Regulatory Networks in the t(4;14) Subgroup

**Fig. 2:**
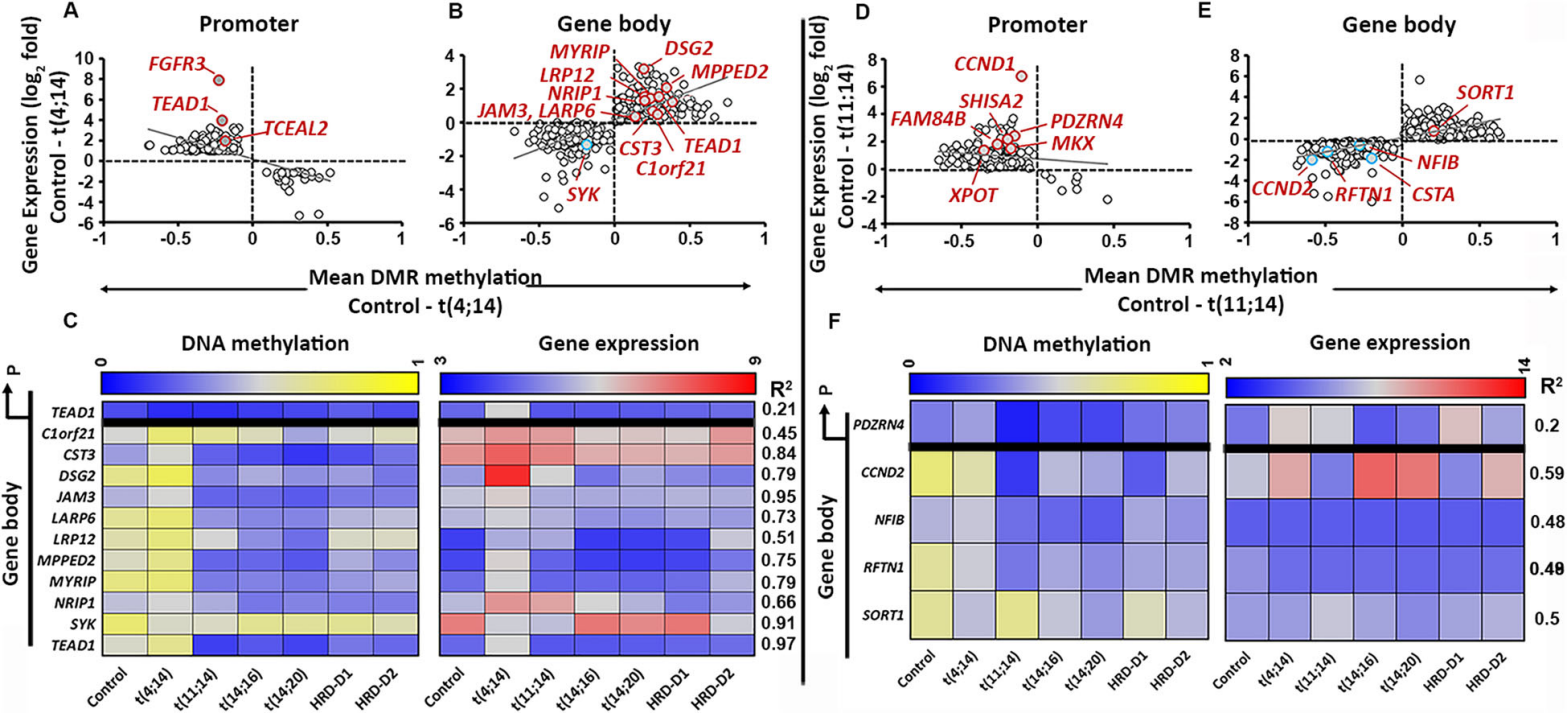
Methylation-expression correlation in the t(4;14) and t(11;14) subgroups. DNA methylation level (horizontal; median values) at DMRs vs. expression (vertical; log2 values) of nearest genes were plotted for promoter (**a**) and gene body (**b**) in t(4;14) and t(11;14) (**d**, **e**) subgroups compared to the control. DMR methylation level vs. expression (based on correlation r^2^>0.2, excluding control) of selected biologically relevant GEMs in MM were represented as heat maps for the t(4;14) (**c**) and t(11;14) subgroup (**f**).

A pairwise comparison in median DMR-methylation (MDM) between control and the t(4;14) subgroup revealed 538 DMRs at promoters and 3798 DMRs within the gene body. Of these DMRs, 671 correlated to the expression of their nearest gene (**Fig.****2****a and b; Supplementary Table****7**). Of the total, we also identified 18 (3.4%) GEMs, reported previously in relation to their oncogenic importance in MM (**Supplementary Table****6**). We identified key t(4;14)-associated genes out of 257 GEMs including *C1orf21, CST3, DSG2, JAM3, LARP6, LRP12, MPPED2, MYRIP,* or *NRIP1,* in which the median hypermethylation at the gene-body DMRs was correlated to their over-expression. Also, 305 GEMs, including *SYK,* contained hypomethylated gene body DMRs and were found to be under-expressed [28]. Heat-maps containing DMR-methylation and expression of corresponding genes, based on the correlation coefficient (r^2^>0.2) across the MM subgroups, were plotted (**Fig.****2****c)**.

**Fig. 3:**
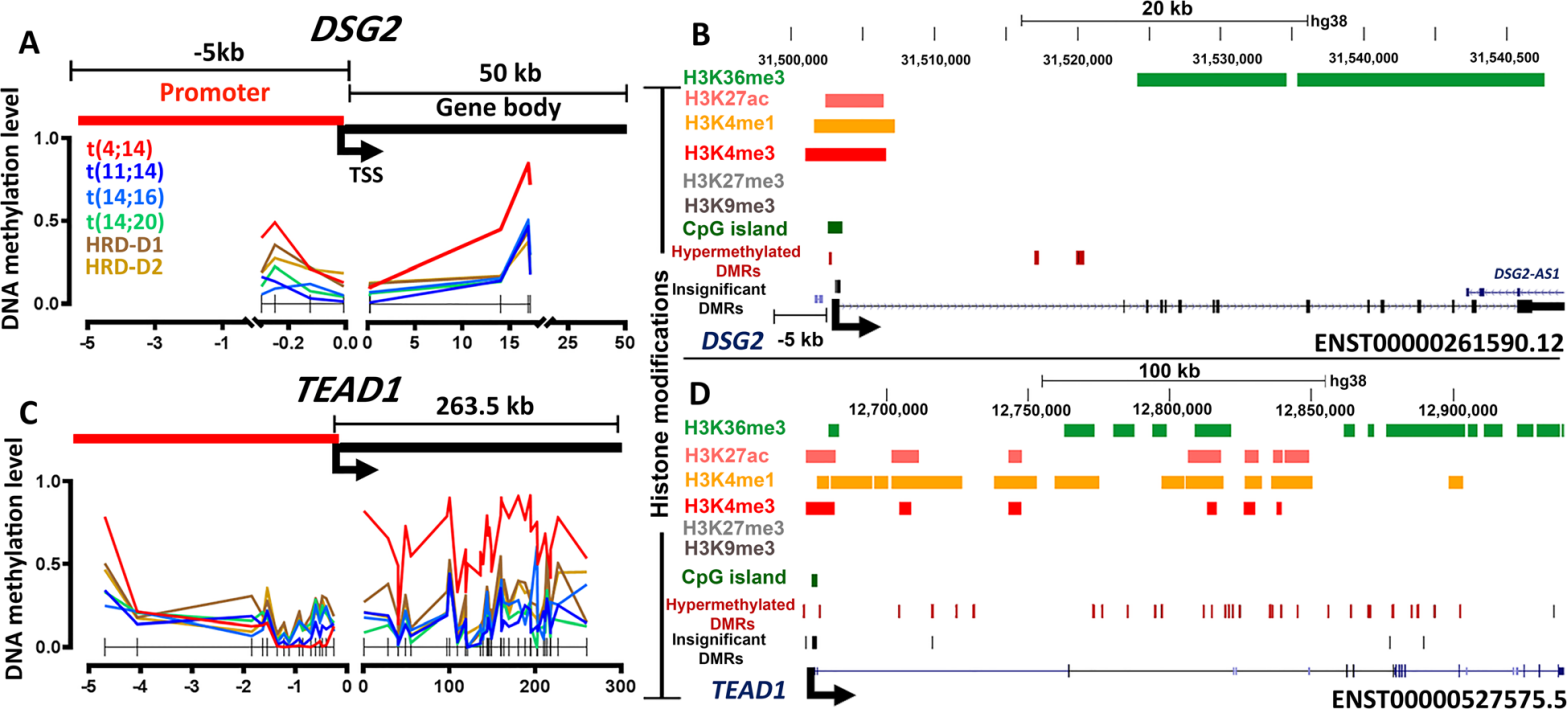
DNA methylation and histone modification profile of *DSG2* and *TEAD1* in the t(4;14) subgroup. (**a**) DNA methylation distribution along the DMRs (p<0.05) at the promoter (up to 5 kb upstream of the TSS) and gene body of the *DSG2* transcript ENST00000261590.12. (**b**) Observed enrichment of activating histone marks, such as H3K4me1, H3K4me3 or H3K27ac at the promoter and H3K36me3 at the gene body of *DSG2* in a t(4;14) cell line. (**c**) DNA methylation distribution along the DMRs (p<0.05) of the *TEAD1* transcript ENST00000527575.5. (**d**) Observed enrichment of activating histones as the major marks, overlapped with *TEAD1* DMRs.

Next, we demonstrated the epigenetic impact on the expression of two chosen t(4;14) GEMs, *DSG2* and *TEAD1* [3, 29]. Three hypermethylated DMRs at the *DSG2* promoter, where the MDM changed from 27% in the control to 39% in the t(4;14) group (p<0.05), enriched with H3K4me1, H3K27ac, and H3K4me3 activating marks were identified. In contrast, five hypermethylated DMRs at the gene body, with an MDM change from 34% in the control to 45% in t(4;14) (p<0.05) samples, were enriched with H3K36me3 (**Fig.****3****a and b)**. In contrast, *TEAD1* contained 16 hypomethylated DMRs, where the MDM changed from 14% in control to 1.4 % in t(4;14) (p<0.05) samples at the promoter and 31 hypermethylated DMRs, where the MDM changed from 54% in control to 68% in t(4;14) (p<0.05) samples at the gene body (**Fig.****3****c**). *TEAD1* gene body DMRs overlapped with several activating histone marks (**Fig.****3****d**). Differential expression of *DSG2* in t(4;14) is thus epigenetically supported by gene body hypermethylation and enrichment in H3K36me3, while *TEAD1* expression was related to the canonical DNA methylation-expression relationship at the promoter and gene-body.

Finally, we predicted the involvement of the t(4;14) GEMs in KEGG annotated pathways (**Supplementary Table****8**). GEMs with increased expression due to promoter hypomethylation were associated with spliceosome assembly or oxidative phosphorylation pathways, while upregulated GEMs with hypermethylation at the gene body were associated with mTOR, Rap1, PI3k-AKt, or MAPK pathways (**Supplementary Table****8**).

### Epigenetic Transcriptional Regulatory Networks in the t(11;14) Subgroup

**Fig. 4:**
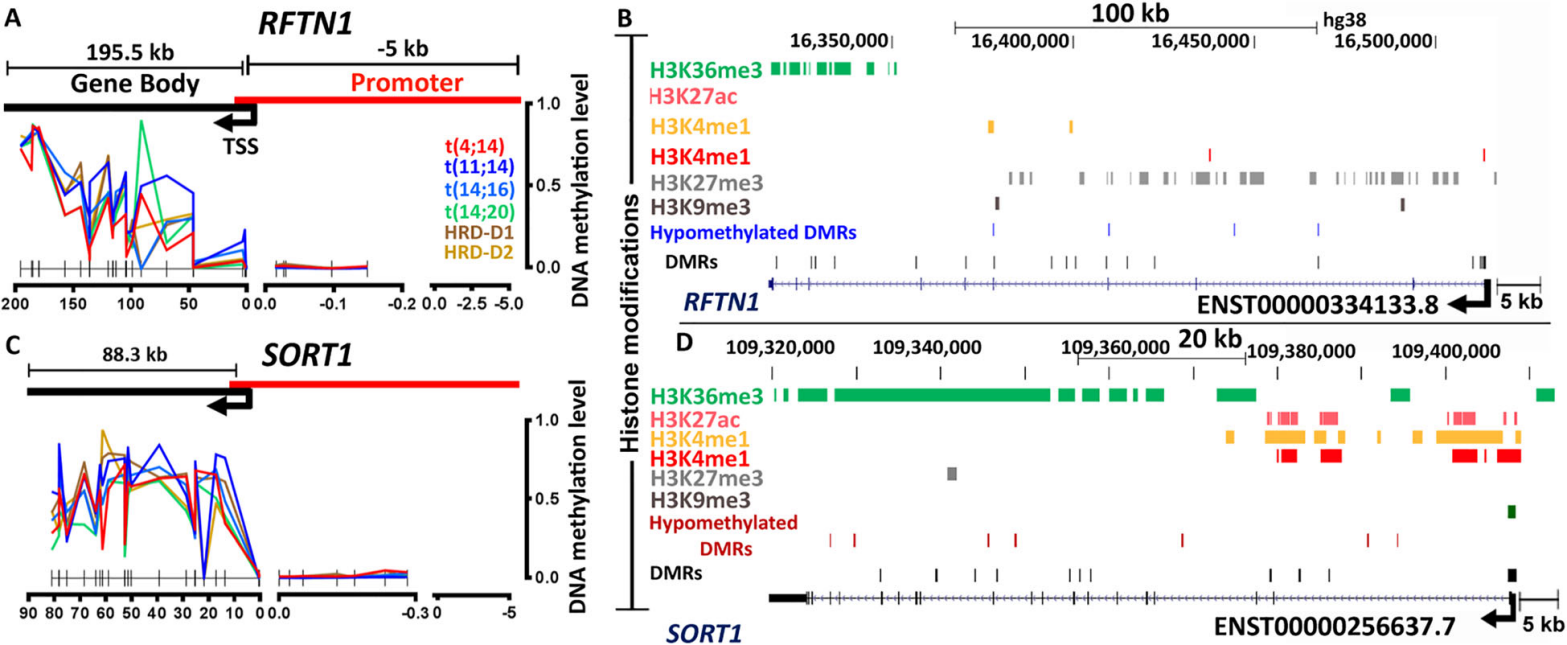
DNA methylation and histone modification profile of *RFTN1* and *SORT1* in the t(11;14) subgroup. (**a**) DNA methylation distribution along the DMRs (p<0.05) at the promoter (up to 5 kb upstream to the TSS) and gene body of the *RFTN1* transcript ENST00000334133.8. (**b**) Observed enrichment of repressive H3K27me3, overlapped with hypomethylated DMRs at the gene-body. (**c**) DNA methylation distribution along the DMRs (p<0.05) of the *SORT1* transcript ENST00000256637.7. (**d**) Observed enrichment of activating histones such as the H3K36me3, overlapped with *SORT1* hypermethylated DMRs at the gene body. Histone marks were obtained from a t(11;14) cell line.

We identified 7510 DMRs and 949 GEMs (**Supplementary Table****9**), including 18 genes (1.8%) that were previously reported as key oncogenes in the t(11;14) subgroup (**Supplementary Table****6**). For example, *CCND1*, *FAM84B*, *MKX*, *PDZRN4*, *SHISA2*, and *XPOT* contain hypomethylated DMRs at the promoters and were over-expressed; while*, CCND2*, *NFIB*, *and RFTN1* contain hypomethylated DMRs at the gene body and were under-expressed (**Fig.****2****d and e)** . We further narrowed down to 5 GEMs including *PDZRN4*, *CCND2*, *NFIB*, *RFTN1*, and *SORT1* based on the level of methylation-expression correlation (r^2^>0.2) (**Fig.****2****f**). The epigenetic alterations on *RFTN1* and *SORT1* expression were investigated in the subgroup [30]. We did not observe any significant difference in methylation across five DMRs at the *RFTN1* promoter. However, the majority of the gene body DMRs were hypomethylated in t(11;14) (MDM: 19%), compared to controls (MDM: 69%) (**Figs.****2****f and****4****a**). We also observed H3K27me3 repressive marks across the gene body (**Fig.****4****b**) using U266 cell line data. This two-tier combinatorial epigenetic effect is correlated to under-expression of the gene. In contrast, *SORT1* was overexpressed and contained hypermethylated DMRs (MDM changed by 4% from control) across the gene body (**Fig.****4****c**) and overlapped with activating histone marks (**Fig.****4****d**).

Overexpressed t(11;14) GEMs containing hypomethylated promoters or hypermethylated body are involvement in MAPK signaling, focal adhesion, oxidative phosphorylation, microRNA metabolism, or cancer related cell cycle pathways. In contrast, under-expressed GEMs were predicted to be involved in cell adhesion molecules, JAK-STAT signaling, or calcium signaling pathways (**Supplementary Tables****10**).

### Epigenetic Transcriptional Regulatory Networks In maf Subgroups

**Fig. 5:**
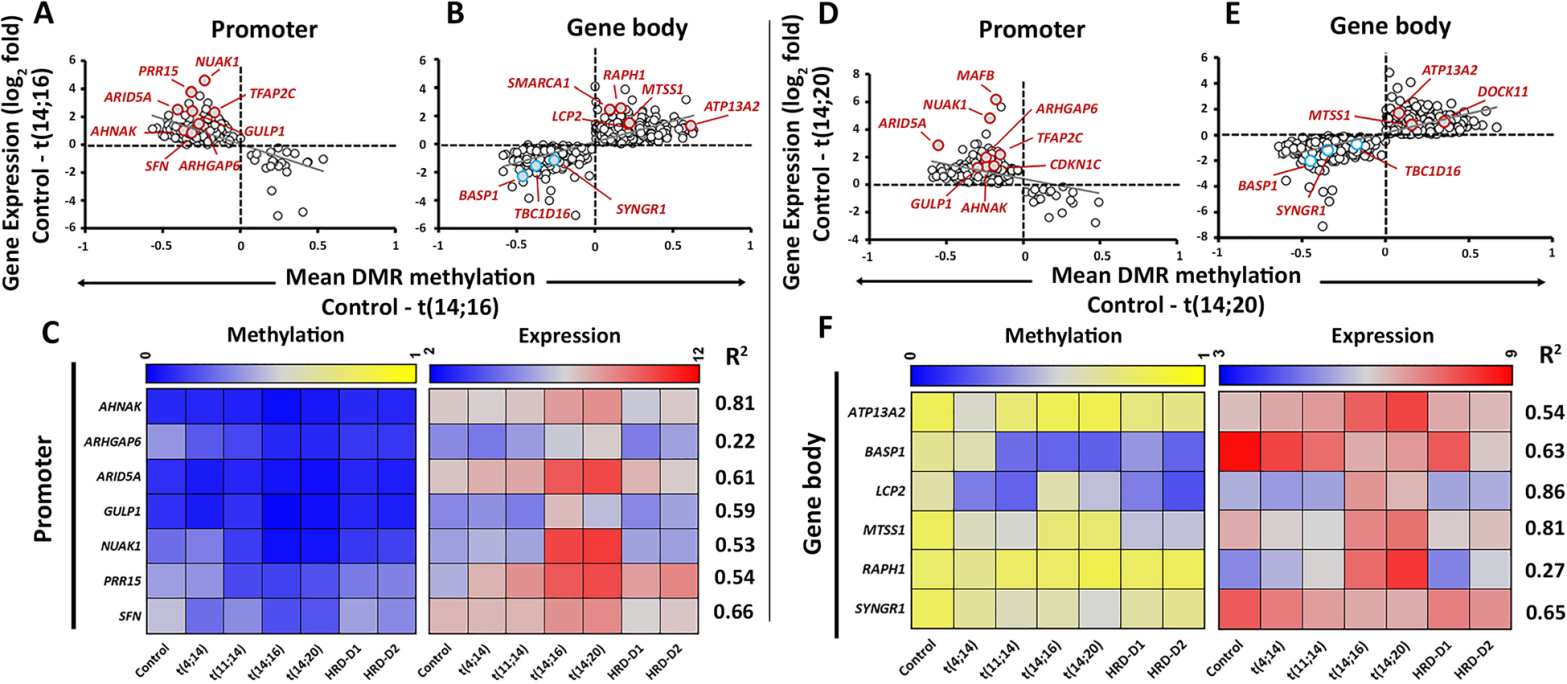
Methylation-expression correlation in the t(14;16) and t(14;20) subgroups. DNA-methylation level (horizontal; median values) at DMRs vs. expression (vertical; log2 values) of nearest genes were plotted for promoter (**a**) and gene body (**b**) in t(14;16) and t(14;20) (**c**, **d**) subgroup. Median DMR methylation level vs. expression (based on correlation r^2^>0.2, excluding control) of selected biologically relevant GEMs in t(14;16) (E) and t(14;20) (F) subgroups.

In the maf subgroups, t(14;16) and t(14;20), we identified 517 mutually shared GEMs, including 28 genes, which are specifically overexpressed in this subgroup (**Supplementary Table****11 and 12**). For example, we observed a negative correlation of MDM at the promoters with increased expression of 208 GEMs in t(14;16) and 251 GEMs in t(14;20) subgroups. These GEMs included previously known oncogenes such as *ARHGAP6*, *AHNAK*, *ARID5A*, *GULP1*, *NUAK1*, and *PRR15* (**Figure****5****a and c)** in the maf subgroups. Additionally, *BASP1* and *SYNGR1* were identified among the hypomethylated and under-expressed genes [n=404 in t(14;16) and n=504 in t(14;20)], and *MTSS1* among the hypermethylated-overexpressed GEMs [n=385 in t(14;16) and n=427 in t(14;20)] (**Fig.****5****b and d**). We also observed t(14;16)-specific GEMs, such as *PRR15* and *SFN*, or t(14;20)-specific GEMs, such as *CDKN1C*, at the promoter.

**Fig. 6:**
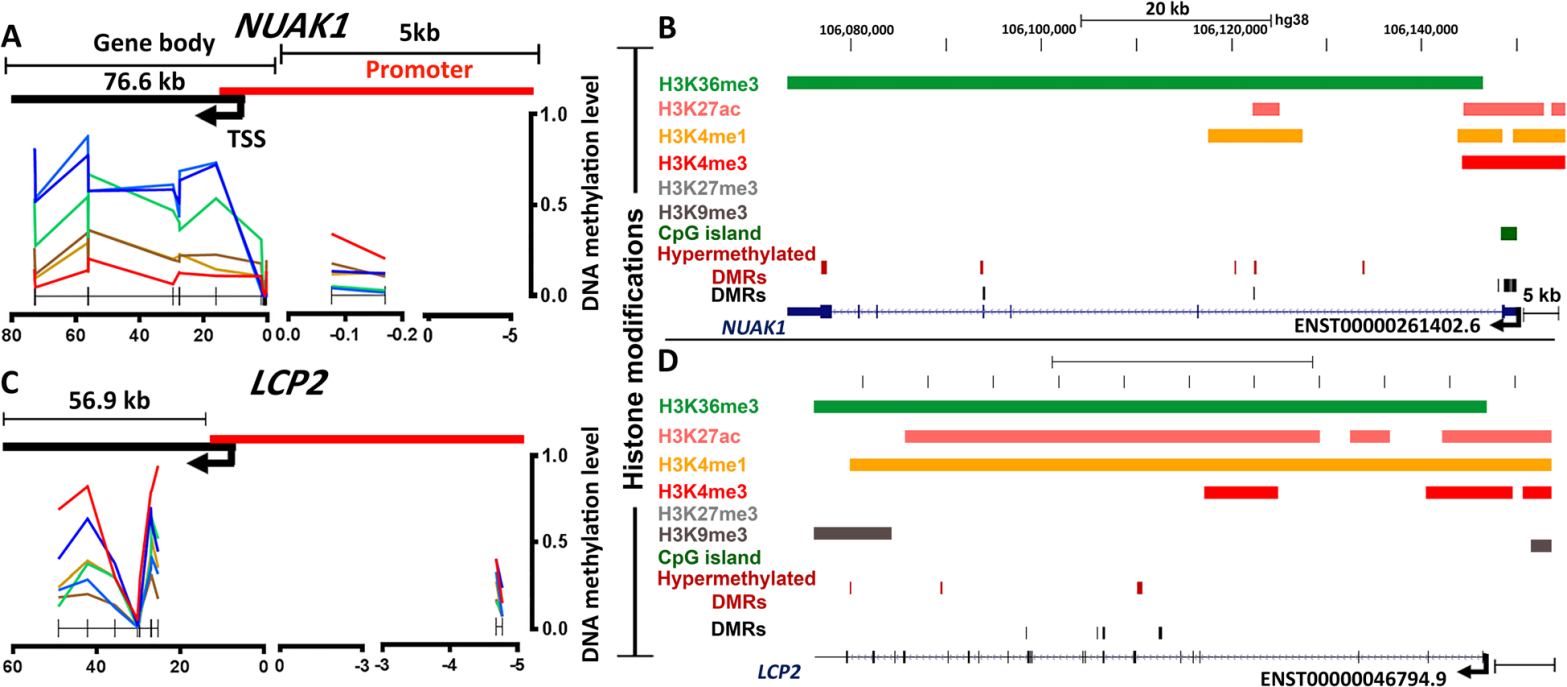
DNA methylation and histone modification profile of *NUAK1* and *LCP2* in the t(14;16) and t(14;20) subgroups. (**a**) DNA methylation distribution along the DMRs (p<0.05) at the promoter (up to 5 kb upstream to the TSS) and gene body of the *NUAK1* transcript ENST00000261402.6. (**b**) Observed enrichment of activating H3K4me1, H3K4me3 or H3K27ac marks, overlapped with hypomethylated DMRs and H3K36me3 marks overlapped with hypermethylated DMRs at the gene body of *NUAK1* in a t(14;16) cell line. (**c**) DNA methylation distribution along the DMRs (p<0.05) of the *LCP2* transcript ENST00000392050.2. (**d**) Observed enrichment of several activating histones marks including H3K36me3, H3K27ac, or H3K4me1, overlapped with hypermethylaated DMRs at the gene body.

Next, we focused on fifteen mutually shared GEMs, of which expression of 8 genes corresponded to the MDM at the promoter, while the remaining GEMs were correlated with the MDM at the gene body (**Fig.****5****e-f; Supplementary Table****6**). Additionally, we examined the DNA methylation and histone level changes in two key GEMs in the maf subgroups [3, 31], namely *NUAK1* and *LCP2*. *NUAK1* was over-expressed and contained two hypomethylated DMRs [MDM reduced to 22% in t(14;16); 21% in t(14;20), compared to control (MDM: 25%; p<0.05)] at the promoter. The gene contained relatively hypermethylated DMRs at the gene body (**Fig.****6****a**), where the MDM increased by 13% in t(14;16) and 5% in t(14;20), compared to control (MDM: 43%; p<0.05). Enrichment of the H3K36me3 throughout the gene body and H3K4me1, H3K27ac, and H3K4me3 marks at the promoter of *NUAK1* were also observed (**Fig.****6****b**). In contrast, *LCP2* was over-expressed [4.6-fold in t(14;16) and 4.8-fold in t(14;20) subgroup; p<0.05] probably due to the presence of hypermethylated DMRs [MDM changed by 9% in t(14;16) and 7% in t(14;20) respectively, compared to the control (MDM: 32%)(p<0.05)] (**Fig.****6****c)** and enrichment of activating histone marks across the gene body **(Fig.****6****d**).

Finally, the hypomethylated-overexpressed GEMs at the promoters in t(14;16) were predicted to be involved in ribosome biogenesis pathway, while hypomethylated- under-expressed GEMs at the body in the Rap1 signaling pathway, or neuroactive ligand-receptor interactions (**Supplementary Table****13)**. The t(14;20)-specific hypomethylated- under-expressed GEMs at the body were predicted to be involved in Rap1, sphingnolipid metabolism, and galactose metabolism (**Supplementary Table****14****)** pathways. Additionally, we identified involvement of maf-GEMs in several common molecular functions such as nuclear biosynthesis and regulation of cell cycle machinery (**Supplementary Table****13 and 14****)**.

### Epigenetic Transcriptional Regulatory Networks In Hyperdiploid Subgroups

**Fig. 7:**
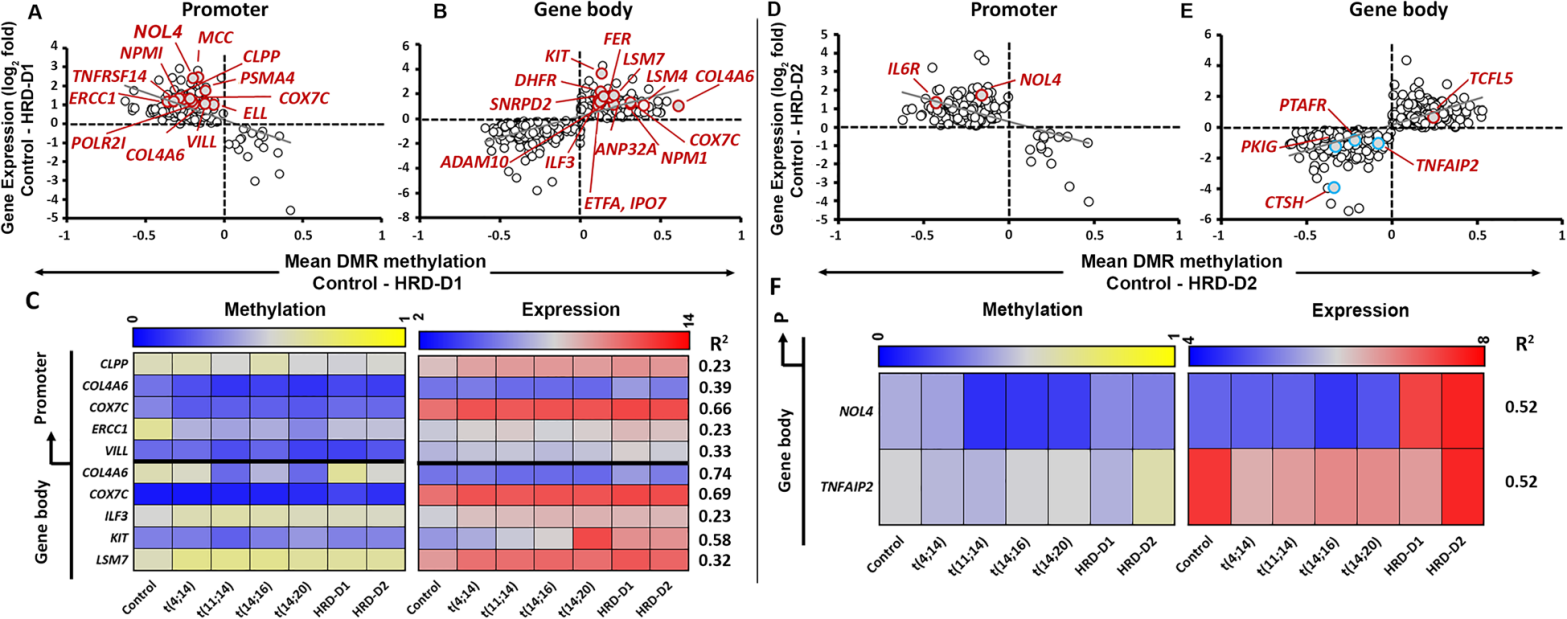
Methylation-expression correlation the hyperdiploid subgroups. DNA-methylation level (horizontal; median values) at DMRs vs. expression (vertical; log2 values) of nearest genes were plotted for promoter and gene body in HRD-D1 (**a**) and HRD-D2 (**b**) subgroups. DMR methylation level vs. expression (based on correlation r^2^>0.2, excluding control) of selected biologically relevant MM GEMs were represented as heat maps for HRD-D1 (**c**) and HRD-D2 subgroups (**d**).

We identified 5605 DMRs, corresponding to 777 GEMs (**Supplementary Table****15**) in the HRD-D1 subgroup, and 4672 DMRs in the HRD-D2 subgroup, corresponding to 662 GEMs (**Supplementary Table****16****).** We identified n=55 (6.4%) GEMs amongst HRD-D1, but a relatively small number of genes (n=3) for the HRD-D2 GEMs (**Supplementary Table****6****)** that have been reported in literature [30]. For instance, we identified some of the well-known over-expressed genes of the HRD-D1 subgroup, such as *COL4A6*, *COX7C*, *ELL*, *ERCC1*, *MCC*, *NDUFS7*, and *NOL4* among 228 genes containing hypomethylated DMRs at the promoter (**Fig.****7****a**). Similarly, of the known over-expressed genes in HRD-D2 subgroup, we observed *NOL4* and *IL6R* among the hypomethylated over-expressed genes (n=194) at the promoter. *CTSH*, *PKIG*, and *TNFAIP2* appeared among the hypomethylated under-expressed cluster (n=288), while *TCFL5* appeared among the hypermethylated over-expressed cluster (n=209) at the gene body in HRD-D2 subgroup (**Fig.****7****b**).

We predicted that over-expressed GEMs with hypomethylation at promoters in HRD-D1 were involved in ribosome biosynthesis, while suppressed GEMs containing hypermethylation at the gene body were involved in PI3K-Akt or Rap1 signaling pathways, cell adhesion molecule regulation, or infection related responses (**Supplementary Table****17****)**. The over-expressed GEMs containing hypermethylated bodies in the HRD-D2 subgroup were predicted to be involved in spliceosome assembly. In contrast, hypomethylated under-expressed GEMs were involved in Rap1 signaling, neuroactive ligand-receptor interaction or similar to infection mediated responses (**Supplementary Table****18**)**.**

### DNA Methylation Correlates with *CCND2* Expression But Not with *CCND1* Expression.

**Fig. 8:**
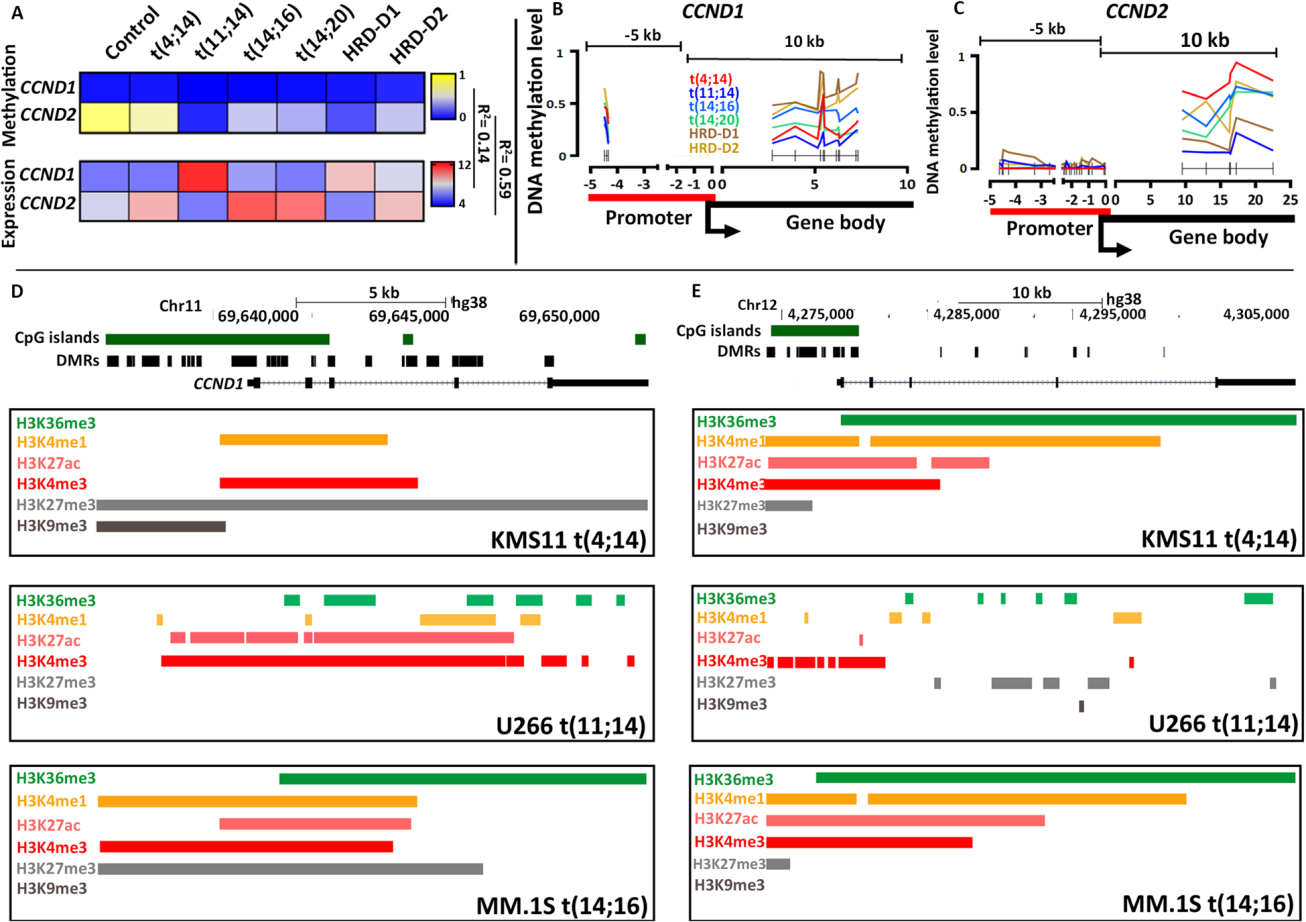
Distribution of DNA methylation and histone modifications of *CCND1* and *CCND2* in NDMM. (**a**) Median DMR methylation level vs. expression correlated for *CCND2* (r^2^=0.59), but not for *CCND1* (r^2^=0.14) across the subgroups. DNA methylation levels across *CCND1* (**b**) and *CCND2* (**c**) for each subgroup*.* (**d**) Chromatin marks at *CCND1* (**d**) and *CCND2* (E) in three different cells lines representing the t(4;14), t(11;14) and t(14;16) subgroups.

We also examined the epigenetic regulation of *CCND1* and *CCND2*, two key cell cycle regulators that are often used to define the genomic subgroups of MM. *CCND1* is highly expressed in the t(11;14) due to the proximity of the translocated *IGH* SE and also in the HRD-D1 subgroup due to gain of chromosome 11 (**Fig.****8****a**). Conversely, the t(4;14), t(14;16), t(14;20), and HRD-D2 have high expression of *CCND2* (**Fig.****8****a**) through an indirect, ill-defined activation route.

Comparing these two cyclin genes we showed that expression is not correlated to DNA methylation for *CCND1* (r^2^=0.14) but is highly correlated for *CCND2* (r^2^=0.59, **Fig.****8****b**). For *CCND1*, as expected the t(11;14) have the highest spike in expression but also have the lowest levels of gene body methylation (**Fig.****8****b**), which would normally be associated with low expression levels. There was no correlation between promoter or gene body methylation and *CCND1* expression (r^2^=0.066 and 0.0382, respectively). However, the expression of *CCND1* can be explained at the histone level, where the U266 cells with a t(11;14) retain high levels of activating marks (H3K36me3, H3K27ac, and H3K4me3) and do not have repressive marks (H3K27me3 or H3K9me3), presumably due to the presence of the translocated *IGH* locus SE, **Fig.****8****e**.

For *CCND2*, there was a high correlation between DNA methylation and expression both overall (r^2^=0.59) and at the promoter and gene body (r^2^=-0.671 and 0.645, respectively). The t(11;14) and HRD-D1 subgroups, which have low *CCND2* expression, had the highest promoter methylation levels and the lowest gene body methylation levels, which is consistent with low expression. The other subgroups, with high *CCND2* expression, all had low or no methylation at the promoter and high levels of gene body methylation, consistent with high expression, **Fig.****8****c**. In addition, in the *CCND2* expressing cell lines KMS11 (**Fig.****8****g**) and MM1.S (**Fig.****8****i**) there was enrichment for active histone marks along the promoter and gene body, which were not present in the t(11;14) containing U266 cells (**Fig.****8****h**). Therefore, *CCND2* expression is highly coordinated between DNA methylation and active histone marks, which is not seen with *CCND1*. It has previously been shown that *CCND2* has a weak super-enhancer in MM1.S cells [20] but the reason for the presence of the super-enhancer is unclear. It may be that the changes in DNA methylation are a result of the active chromatin or vice versa.

### The Functional Impact of CTCF and SEs at Overlapping DMRs on Gene-overexpression.

Earlier literature suggest that altered DNA methylation in the gene regulatory SE-elements may influence over-expression of the target gene or gene-cassette by forming a loop with the aid of CTCF TFs (**Supplementary Figure****8**) [32]. With this in mind, we investigated the potential overlap between the DMRs and SEs in conjunction with CTCF binding sites in NDMM subgroups. We identified 677 SE-DMRs, based on a ChIP-sequence data for BRD4 in the MM1.S cell line [20], or 4,791 CTCF-DMRs from CTCF binding in the Delta47 cell line [19], matched to the overlapping GEM-DMRs of the NDMM patients. On average, we found 65% of SE and 82% of CTCF sites overlapped with hypomethylated DMRs, while the remaining were overlapped with hypermethylated DMRs (**Supplementary Table****20**). Hierarchical clustering using the DMRs overlapping with SE-CTCF sites generated three major SE-DMR or SE-CTCF modules across the promoter, body, and IGRs. These modules contained only differentially hypomethylated, hypermethylated, or a combination of both (**Supplementary Figure****9**) hyper/hypo-DMRs in the subgroups compared to control.

**Fig. 9:**
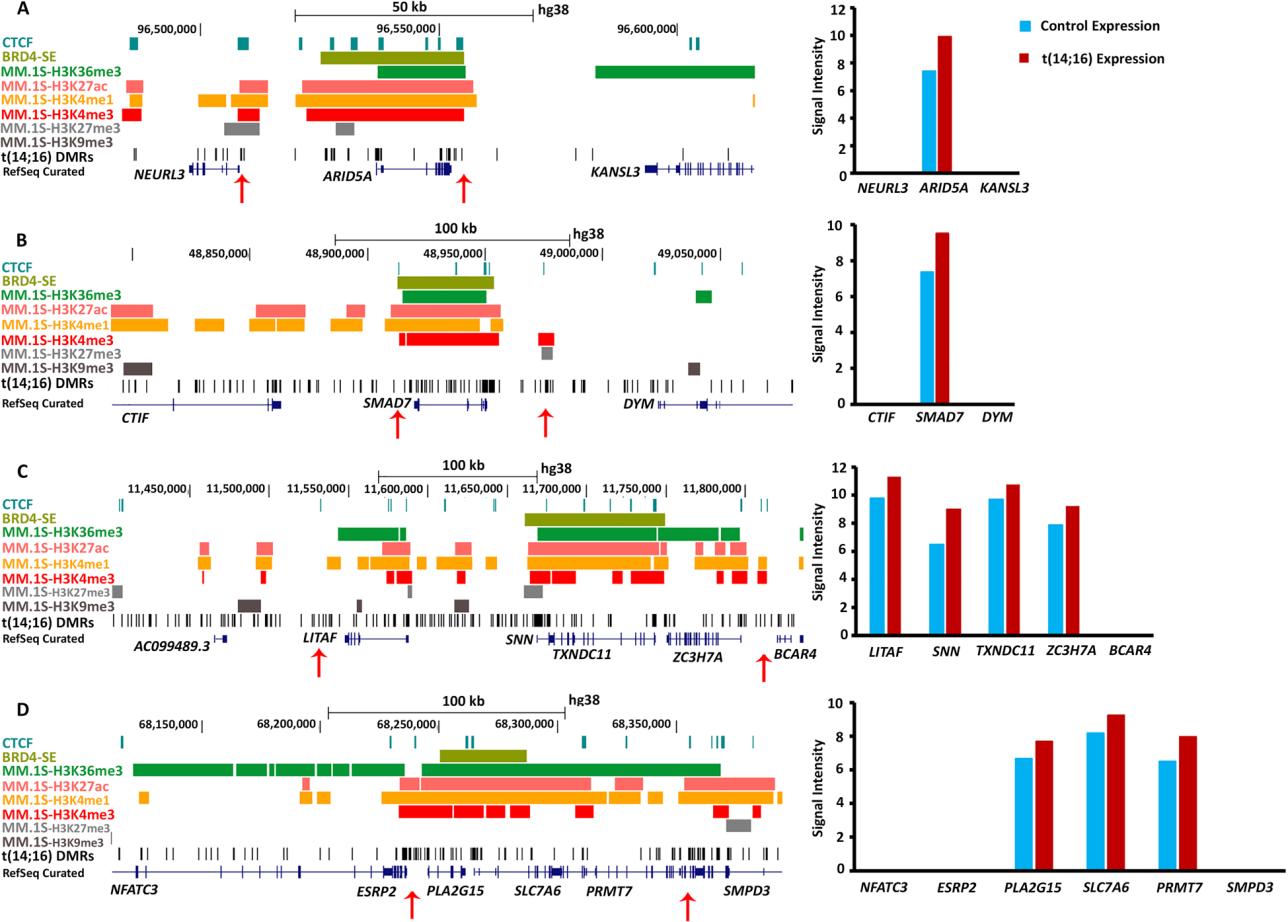
Super-enhancer control of gene-expression in MM. Overlapping of super-enhancer (SE)-CTCF loops to the hypomethylated DMRs of the over-expressed (log2 scale) GEMs in t(14;16). (**a**) *ARID5A* is 5.74-fold overexpressed, while (**b**) *SMAD7* is 4.44-fold over-expressed and are predicted to be under control of a SE-CTCF loop. The surrounding genes do not show over-expression. (**c**) An over-expressed gene cassette containing *LITAF* (2.94-fold increase), the GEM, *SNN* (5.6-fold increase), *TXNDC11* (2.1-fold increase), and *ZC3H7A* (2.55-fold increase), spanning a 50 kb region or (**d**) another gene cassette containing the GEM *PLA2G15* (4.11-fold increase), *SLC7A6* (4.28- fold increase), and *PRMT7* (6.82-fold increase), spanning over 20 kb region were identified in the t(14;16) subgroup. Red arrows show the putative boundaries of the SE-CTCF loops.

Furthermore, we demonstrate the impact of SE-CTCF loops at the H3K27ac enriched DMRs that regulate overexpression of individual or cassettes of GEMs [3, 29] in the t(14;16) subgroup. The promoter or gene-body of these GEMs were either partially overlapped with the SE elements or located within the overlapping SE-loops [32]. We identified eighteen such hypomethylated and over-expressed (>2 fold) GEMs in the t(14;16) subgroup (**Supplementary Table****20**) to elaborate the combinatorial effect of DNA methylation, histone marks, and SEs on gene expression. For example, TSS and promoter upstream regions of *ARID5A* and *SMAD7* were located within a putative interstitial SE-loop, where the hypomethylated DMRs were overlapped with activating histone marks, in particular H3K27ac (**Fig.****9****a and b**). The epigenomic architecture of these GEMs was associated with a 5.7- and 4.4-fold increase in expression, respectively, which was not seen in immediately adjacent genes, outside of the SE-CTCF loops. We also observed the SE-CTCF regulated over-expression of gene-cassettes. For instance, we identified overexpression of *LITAF*, *SNN*, *TXNDC11,* and *ZC3H7A* spanning over a 200 kb region (**Fig.****9****c**), as well as *PLA2G15, SLC7A6*, and *PRMT7* spanning a 100 kb region (**Fig.****9****d**) under the influence of putative SE-loops. Moreover, *MAF*, the signature oncogene of the t(14;16) subgroup, was 15.5 fold over-expressed in our dataset while promoter and upstream enhancer of *MAF* were partially overlapped to the SE elements (**Supplementary Figure****10**), besides *IgH-SE* overlap to the 3’-end of the gene (data not shown).

## Discussion

In the present study, we have combined alterations in genome-wide DNA methylation and histone modifications with regulatory mechanisms of SE-CTCFs to explain the subgroup-specific differential gene expression dysfunctions in NDMM patients. The GEMs reported here not only serve as the epigenetic biomarker for the early detection of the disease, but also give an idea about successive oncogenic transitions in MM.

Autosomal epigenetic traits in B cells are considered to propagate to the daughter cells in an accumulative pattern through the stages of differentiation [33] which strengthens the importance of differential DNA methylation as a specific predictor of disease progression in MM. We observed that the DNA methylation data points for the top 5% variable DMRs were found to affect >94% of autosomal CpG sites across the molecular subgroups. Furthermore, we show that, except for t(4;14), DNA hypomethylation was prevalent across the major genomic regions including promoters, gene bodies, or IGRs in the remaining MM subgroups compared to age matched controls. The amount of hypermethylation in t(4;14) relative to non-t(4;14) subgroups may be explained by the over-expression of MMSET, an H3K36 methyltransferase [34]. In contrast, global DNA-demethylation in MM subgroups may be attributed to frequent mutations in epigenetic modifiers, especially *DNMT3A* [35], which may create favorable conditions of genome-wide DNA hypomethylation within and outside the CpG islands.

When we combined methylation data at variable CpGs with gene expression, it was found that even though DNA-methylation is inversely correlated to the expression in promoters of the nearest genes, they may be positively correlated to the expression at the gene bodies, as evidenced by the key genes per subgroup. Moreover, regulation of gene expression is not merely influenced by the changes in DNA methylation at promoters or within the gene body, but are tightly linked to the overlapping chromatin modifications, or regulated from the juxtaposed SE-CTCF loops (**Supplementary Table****20**). For instance, we observed a series of GEMs, where the DMRs were differentially hypomethylated at the gene body but remained over-expressed in a particular MM subgroup. These events could be correlated to the effect of the overlapping activating histones or regulatory control from the SEs (**Supplementary Table****21**). At the histone level, the majority of the hypomethylated DMRs overlapped with repressive H3K27me3, while the majority of the hypermethylated DMRs within the gene body overlapped with H3K36me3 and H3K4me1 or H3K4me3 marks. A strong association of these activating histones has already been reported in relation to their regulatory role in the alternative splicing mechanisms in cancer [36]. The preferential occurrence of these histones has also been reported in MM as biomarkers and druggable targets [37–39]. Additionally, loci-specific enrichment of H3K27ac at the DMRs suggest the existence of interstitial SE-like regulators, which may create a transcriptionally active state resulting in over-expression of genes or gene clusters in MM. We found that these acetylated and hypomethylated DMR domains are also the preferential binding sites of BRD4 or MED1. These BRD4/MED1 binding sites are generally demarcated by CTCFs at the termini that presumably form the SE-CTCF loops. SE-CTCF loops spanning the length of an entire gene or gene-cassette lead to the aberrant over-expression of genes.

The most common pathways predicted to be affected by the GEMs of different MM subgroups, were PI3K/AKT/mTOR, MAPK, Rap1 and the cell cycle. These pathways align with the existing literature, where frequent activation of the PI3K/AKT/mTOR pathway [40], or recurrent mutations and aberrant expression of MAPK [41] or Rap1 [42] genes have been reported in MM patients. Interestingly, six GEMs in the t(4;14) subgroup were upregulated and are part of the Rap-guanine nucleotide exchange family, upstream of Rap1. In contrast, a different set of GEMs in the maf and HY subgroups, containing both upstream activators and downstream targets of Rap1 were down-regulated. While the upstream regulators mainly constitute membrane receptor kinases, the downstream GEMs were involved in the cellular adhesion, polarity or migration machinery. Cell-cycle pathway genes were also differentially expressed, including *CCND1*, *CCND2,* and *CDKN2C*. These genes are associated with proliferation and prognosis in MM patients. Therefore, the present study provides deeper insights into the epigenetic control of gene expression, and its involvement in different MM signaling pathways.

We also demonstrated epigenetically controlled expression of *CCND1* and *CCND2* among the MM subgroups. For instance, *CCND1* is known to be overexpressed in the t(11;14) subgroup and occurs through the juxtaposition of the *IgH-SE* next to *CCND1*. This results in an epigenetic sweep of the active histone marks from the IgH locus across *CCND1*. In contrast, *CCND2* is not expressed in the t(11;14) subgroup, and may be explained by the enrichment of repressive H3K27me3 marks, lack of activating H3K4me1 and H3K27ac marks, and hypomethylated DMRs within the gene body of *CCND2*. In contrast, a weak SE signal is present in the t(14;16) cell line MM1.S at the *CCND2* promoter, characterized by H3K27ac, H3K4me3, and BRD4 enrichment. A similar ChIP profile is seen in the t(4;14) cell line KMS11. These active histone marks were found in conjunction with DNA hypermethylation within the gene body of *CCND2*, indicating a possible interaction between the epigenetic states. An interesting hypothesis would be to alter the DNA methylation levels within the body of *CCND2* and determine if this alters the chromatin marks and expression of the gene in these high risk *CCND2* expressing subgroups [43].

## Conclusions

Overall, the present study highlights the existence and impact of epigenetic regulatory networks in determining expression abnormalities in the NDMM subgroups, which play key roles in the onset and development of the disease. We observed that under-expressed GEMs in NDMM subgroups generally contained hypomethylated DMRs enriched with repressive H3K27me3 marks; while the overexpressed GEMs harbored clustered hypomethylation at promoters and hypermethylation within the gene body, interacting with activating histone marks. Additionally, hypomethylated DMRs at the gene body or promoters of certain GEMs were enriched with H3K27ac and marked by the preferential binding of BRD4 that behave as locally active elements. Previously, the effect of recurrent mutations in epigenetic modifiers in relation to the survival of MM patients was reported by our group [35]. The data obtained here will give further insight into the epigenetic mechanism of key genes in MM, and also help in determining the subgroup-specific therapeutic regimen. Currently DNA methylation modifying agents and bromodomain inhibitors such as decitabine and JQ1 respectively, may be used to alter the global aberrant epigenetic landscape of tumors and regulate the abnormal expression of downstream oncogenes.

## Supplementary information

**Additional file 1.** Supplementary Information-1.

**Additional file 2.** Supplementary Information-2.

## Data Availability

'The data that support the findings of this study are available from the authors upon reasonable request and with permission of University of Arkansas for Medical Sciences.

## Abbreviations

ChIP: Chromatin immunoprecipitation
CTCF: CCCTC-binding factor
DMC: Differentially methylated CpG
DMR: Differentially methylated region
eRRBS: Enhanced reduced representation bisulfite sequencing
FDR: False discovery rate
GEP: Gene expression profiling
GO: Gene ontology
IgH: Immunoglobulin heavy chain
IGR: Intergenic region
NDMM: Newly diagnosed multiple myeloma
MM: Multiple myeloma
MGUS: Monoclonal gammopathy of undetermined significance
PC: Plasma cell
SE: Super enhancer
STR: Short tandem repeat
TSS: Transcription start site
