# Supplementary material for: The functional epigenetic landscape of aberrant gene expression in molecular subgroups of newly diagnosed multiple myeloma
Source: J Hematol Oncol. 2020 Aug 6;13:108. doi: 10.1186/s13045-020-00933-y (PMC7409490; doi:10.1186/s13045-020-00933-y)
Supplement: Supplementary file 1 — Additional file 1. Supplementary Information-1. [file 13045_2020_933_MOESM1_ESM.pdf]

The functional epigenetic landscape of aberrant gene expression in molecular subgroups of newly diagnosed multiple myeloma

Supplementary Information

Samrat Roy Choudhury[1], Cody Ashby[1], Ruslana Tytarenko[1], Michael Bauer[1], Yan Wang[1], Shayu Deshpande[1], Judith Dent[1], Carolina Schinke[1], Maurizio Zangari[1], Sharmilan Thanendrarajan[1], Faith E. Davies[2], Frits van Rhee[1], Gareth J. Morgan[2], and Brian A. Walker[1,3*]

Affiliations

[1]Myeloma Center, University of Arkansas for Medical Sciences, Little Rock, AR 72205, USA.
[2]Department of Medicine, NYU Langone Health, New York, NY 10016.
[3]Division of Hematology Oncology, Indiana University, Indianapolis, IN 46202.

Corresponding Author

Prof. Brian A. Walker, Division of Hematology Oncology, Indiana University, Indianapolis, IN, USA. Email: bw75@iu.edu. Phone: +1 317 278 7733.

Supplementary Results

Supplementary Figure 1

Unsupervised principle component analysis (PCA) of eRRBS data containing all the samples of different NDMM subgroups, used in the present study. The proportion of variance for the principle component (PC)-1 is 32%, PC2 is 8% and PC3 is 4%. The red circles demarcates distinct segregation of the control and t(4;14) samples from the remaining MM subgroups, based on their DNA-methylation profile.

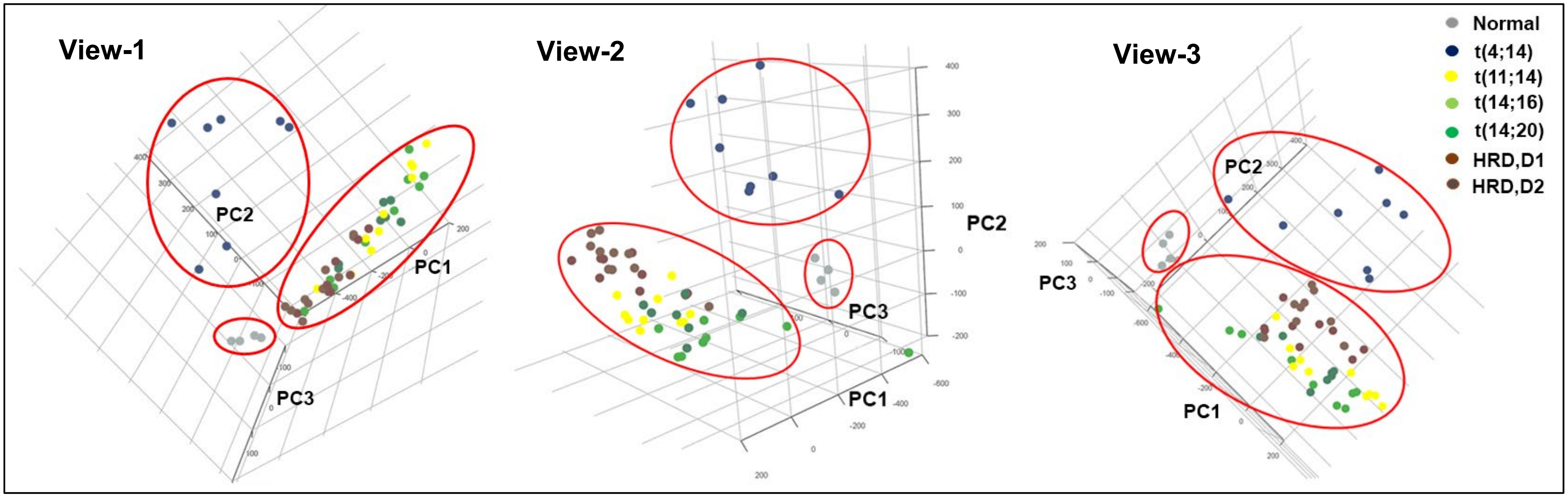

Supplementary Figure 2

(A) The 5% most variable differentially methylated regions (DMRs) in NDMM subgroups, compared to the matched controls were observed, and annotated against the Refseq genomic features. It revealed that the majority of the DMRs were observed in Gene body (51%; 6594 DMRs), followed by intergenic regions (39%; 5050 DMRs), and promoters (10%; 1282 DMRs) **(B)** Based on bootstrapping of the methylation of top 5% most variable DMRs (as obtained from eRRBS), the t(4;14) subgroup formed the nearest cluster to the control. We also observed distinct clustering of the t(14;16) and t(14;20) or HRD-D1 and HRD-D2 subgroups. AU: Approximately unbiased p-value; BP: Bootstrap probability. We measured Euclidian distance of the average clusters (pvclust program of the R package).

(A)

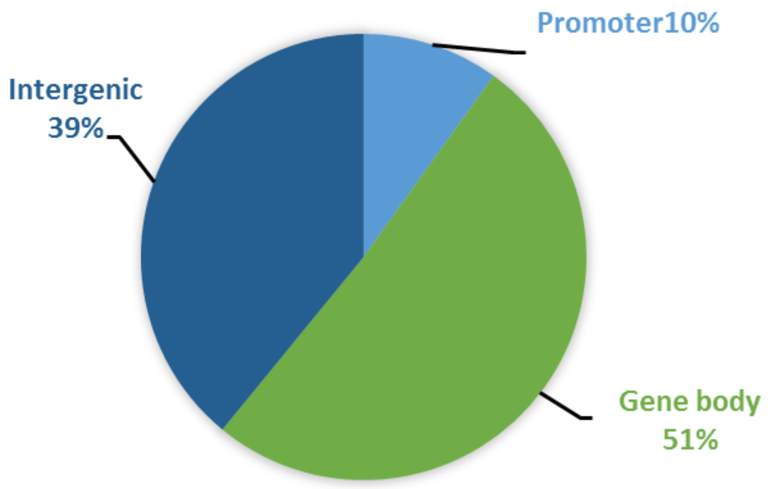

(B)

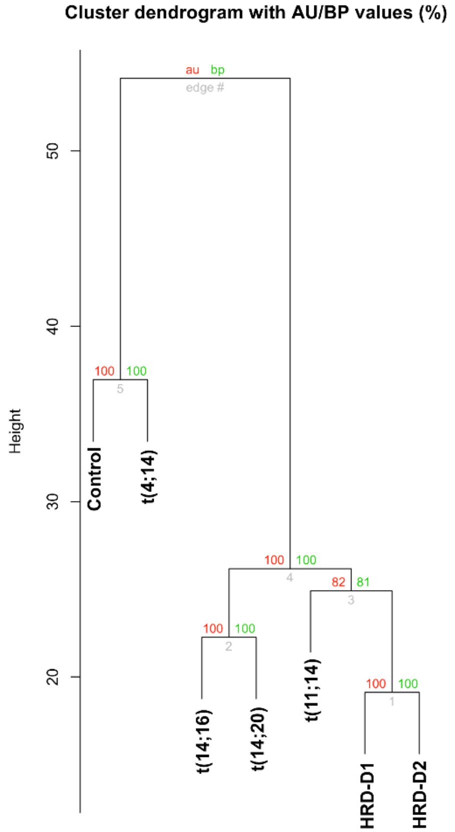

Infinium 850K array was used for determining differentially methylated CpG sites in NDMM subgroups

The distribution of Differentially Methylated CpGs (DMCs) within or beyond DMRs were determined using 850K Infinium methylation arrays in selected samples. The samples from each MM subgroup, analyzed by eRRBS or methylation array, showed high reproducibility between the two methods for these most variable sites (correlation coefficient >91%). PCA analysis based on 850K array showed some heterogeneity between the samples within t(14;16) subgroup (**Supplementary Fig. 3).**

Likewise eRRBS, the highest number of most variable DMCs seen on the arrays were obtained at intergenic regions (42%) and gene bodies (39%) followed by transcription start site (TSS) (10%), 5'-UTRs (6%), 3'-UTRs (2%), and first exons (1%) (**Supplementary Fig. 4A; Supplementary Table 4**). This strengthens the eRRBS data and confirms prevalent distribution of hypomethylated CpGs along the gene body and intergenic regions. This suggest existence of significant epigenetic regulatory elements at gene body that might contribute to the transcription machinery in MM.

The top 5% (n=43,223 unique sites) of most variable DMCs from the methylation array have different hits of CpGs than the eRRBS DMRs, showed similarity in eRRBS DMR-methylation pattern at the major annotated regions (**Supplementary Fig. 4B**). It also showed that the t(4;14) subgroup was relatively hypermethylated, with the t(11;14) subgroup being the most hypomethylated compared to the other NDMM subgroups. We also noticed formation of neighboring clusters among the t(14;16) and t(14;20) or HRD-D1 and HRD-D2 subgroups. Based on the bootstrapping analysis from eRRBS, we observed that the t(11;14) subgroup formed the farthest cluster to the control, as obtained from eRRBS, while in 850 array, we found isolated clustering of t(11;14) from other MM subgroups, but not essentially the most distant cluster from the control.

Supplementary Figure 3

Unsupervised principle component analysis (PCA) of 850k methylation array data of all the samples from different NDMM subgroups, used in the present study. The proportion pf variance for the principle component (PC)-1 is 17%, PC2 is 7%, and PC3 is 5%. The red circles demarcates distinct segregation of majority of the control and t(4;14) samples from the remaining MM subgroups, based on their DNA-methylation profile. PC stands for principle component.

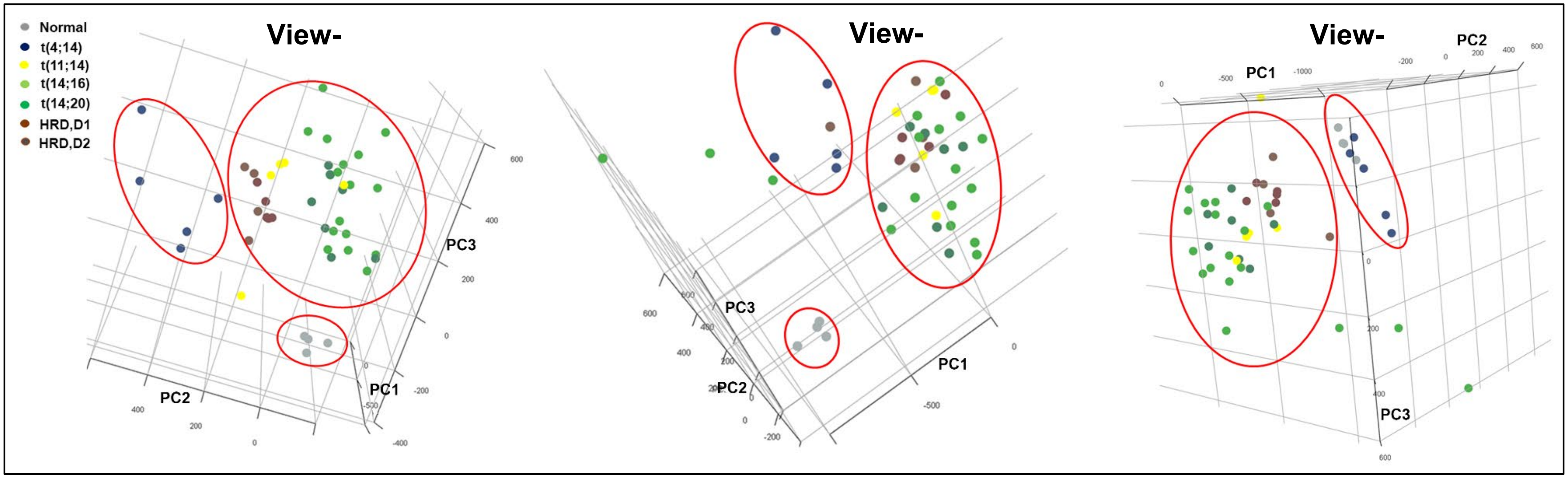

Supplementary Figure 4

(A) Infinium 850k array methylation microarray revealed that majority of differential methylated CpGs (DMCs) were observed at the RefSeq annotated intergenic regions (42%; 17,954 DMCs), followed by gene body (39%; 16,641 DMCs), TSS (10%; 4431 DMCs), 5-UTR (6%; 2638 DMCs), 3'-UTR (2%; 852 DMCs), 1st exon site (1%; 509 DMCs) and exon-bound sites (0%; 198 DMCs). **(B)** The top 5% most variable DMCs formed two distinct and major cluster between the control and MM subgroups. It was also observed that the t(4;14) subgroup was relatively most hypermethylated of all the MM subgroups and formed the closest cluster to the control. In contrast, hyperdiploid groups (HRD) i.e. HRD-D1 and D2 subgroups were the most hypomethylated of all other MM subgroups.

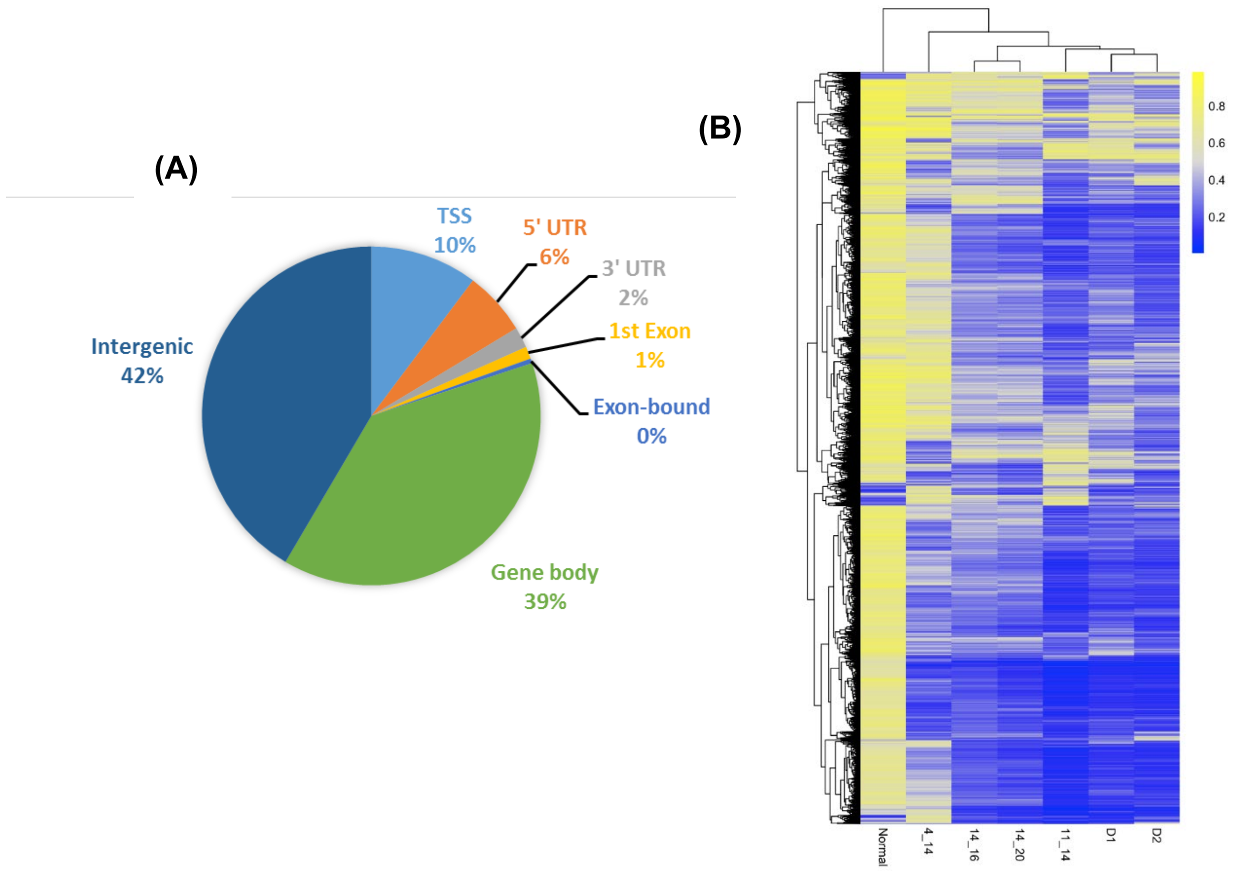

Supplementary Figure 5

Genome-wide differential (FDR<0.05) expression profile in different subgroups of multiple myeloma. We enumerated **(A)** 1483 overexpressed and 695 under-expressed genes in the t(4;14) subgroup, **(B)** 2451 overexpressed and 837 under-expressed genes in the t(11;14) **(C)** 2057 overexpressed and 712 under-expressed genes in the t(14;16) **(D)** 2692 overexpressed and 794 under-expressed genes in the t(14;20) **(E)** 2418 overexpressed and 1035 under-expressed genes in the HRD-D1, and **(F)** 1946 over-expressed and 639 under-expressed genes in the HRD-D2 subgroup. The key genes per MM subgroup were marked. **(G)** Based on bootstrapping of the differential expression homology of top 5% variable genes, we observed that the t(14;16) and t(14;20) subgroups formed the closest cluster to control, followed by t(11;14), t(4;14) and HY subgroups. AU: Approximately unbiased *p*-value; BP: Bootstrap probability. We measured Euclidian distance of the average clusters (pvclust program of the R package). AU: Approximately unbiased *p*-value; BP: Bootstrap probability. We measured Euclidian distance of the average clusters (pvclust program of the R package).

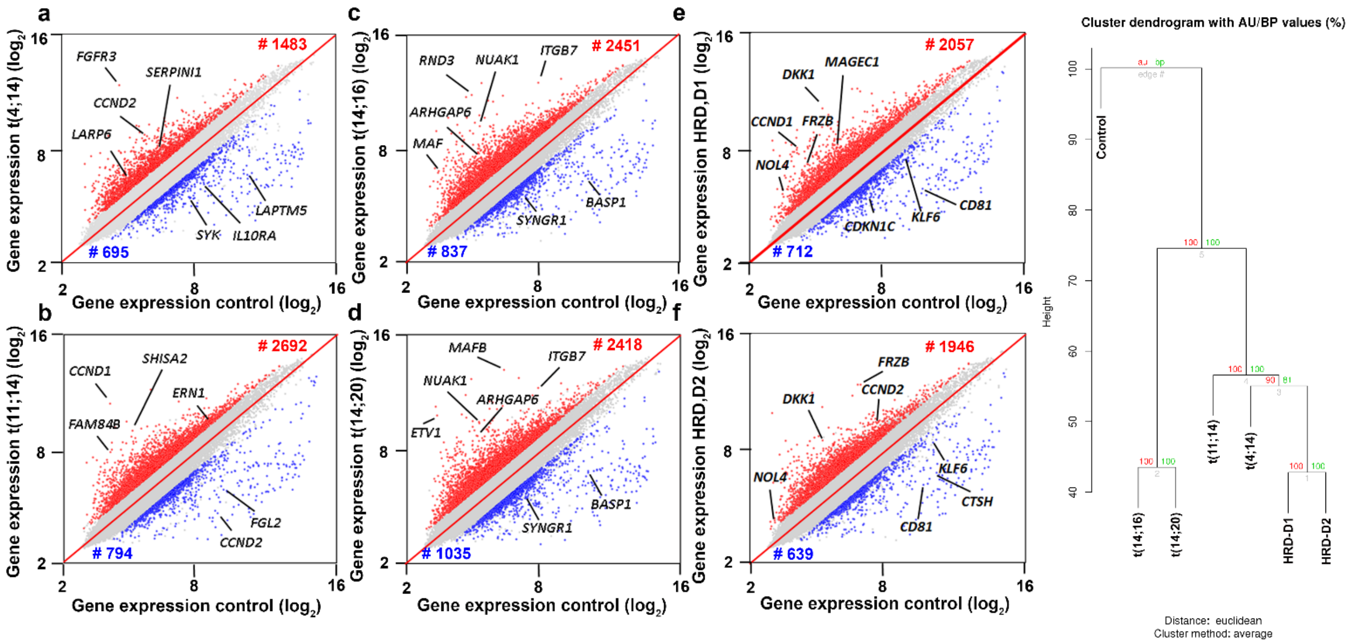

Supplementary Figure 6

Difference in the distribution of hypomethylated epitranscript-DMRs at the promoter **(A)** and body **(B)** or hypermethylated epitranscript-DMRs at the promoter **(C)** and gene body **(D)** across the NDMM subgroups were evaluated using ANOVA

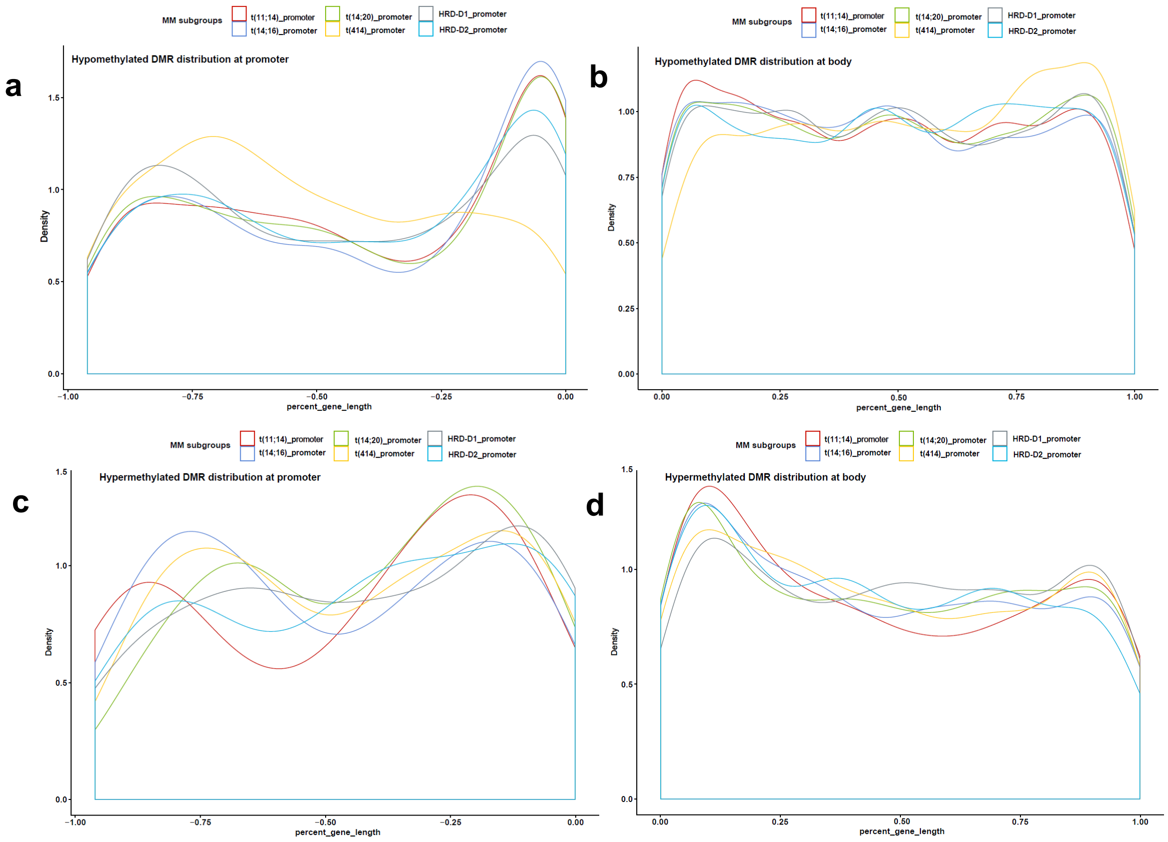

*The P value (Pr) is larger than the F ratio and the inter-group DMR-methylation values for degrees of freedom in the ANOVA table.

	Hypo-DMRs at promoter	Hypo-DMRs at body	Hyper-DMRs at promoter	Hyper-DMRs at body
**Pr (>F) value**	1.55e-09 ***	6.11e-14 ***	0.71	0.114

Supplementary Figure 7A

Depiction of overlap between the hyper/hypo-methylated DMRs in t(4;14) NDMM patients and histone marks from KMS11 cells. Differentially expressed genes in the t(4;14) subgroup, which span these DMR-histone overlapped sites, are shown at the promoter and gene body as both counts and as a percentage.

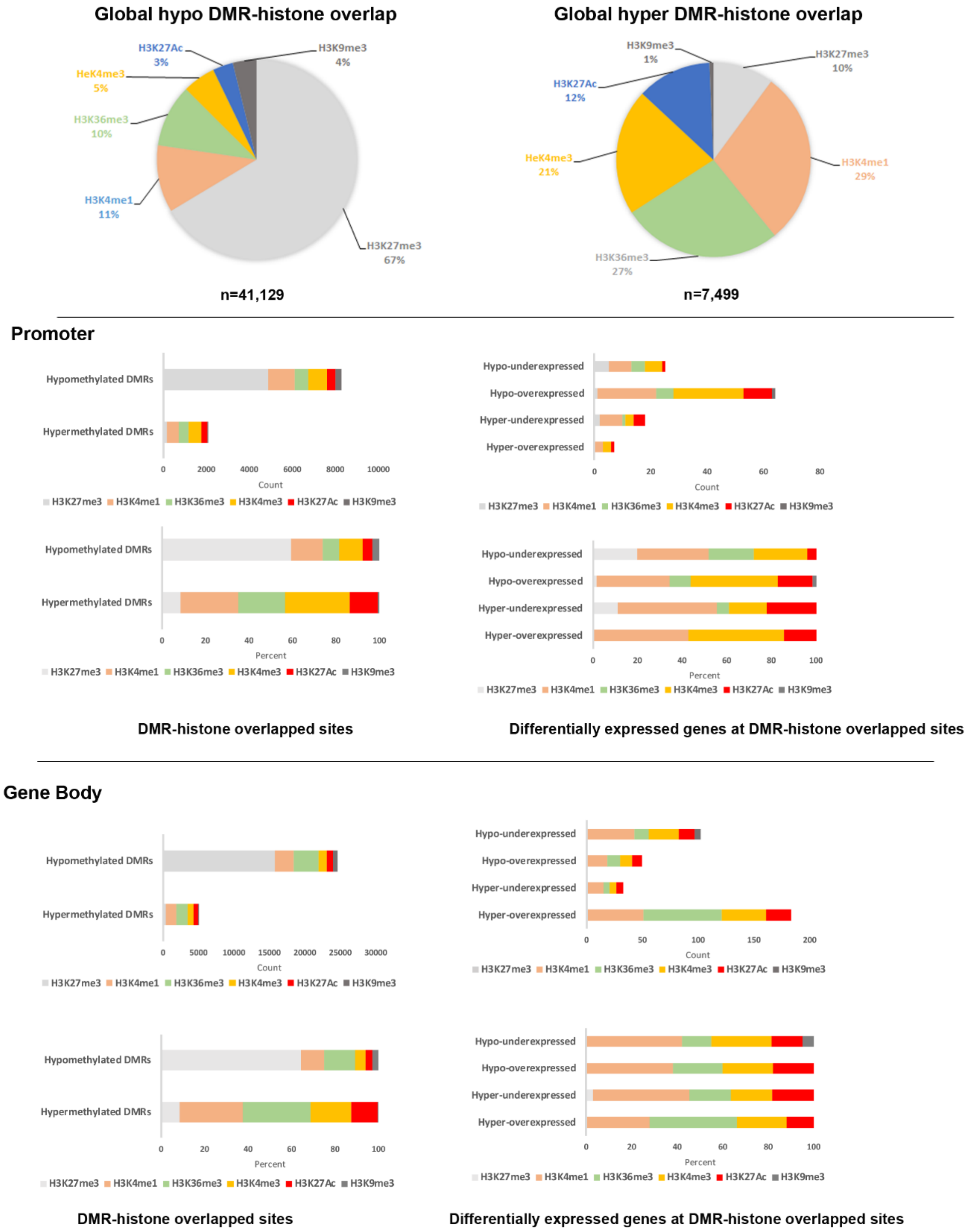

Supplementary Figure 7B

Depiction of overlap between the hyper/hypo-methylated DMRs in t(11;14) NDMM patients and histone marks from U266 cells. Differentially expressed genes in the t(11;14) subgroup, which spans these DMR-histone overlapped sites are shown at the promoter and gene body as both counts and as a percentage.

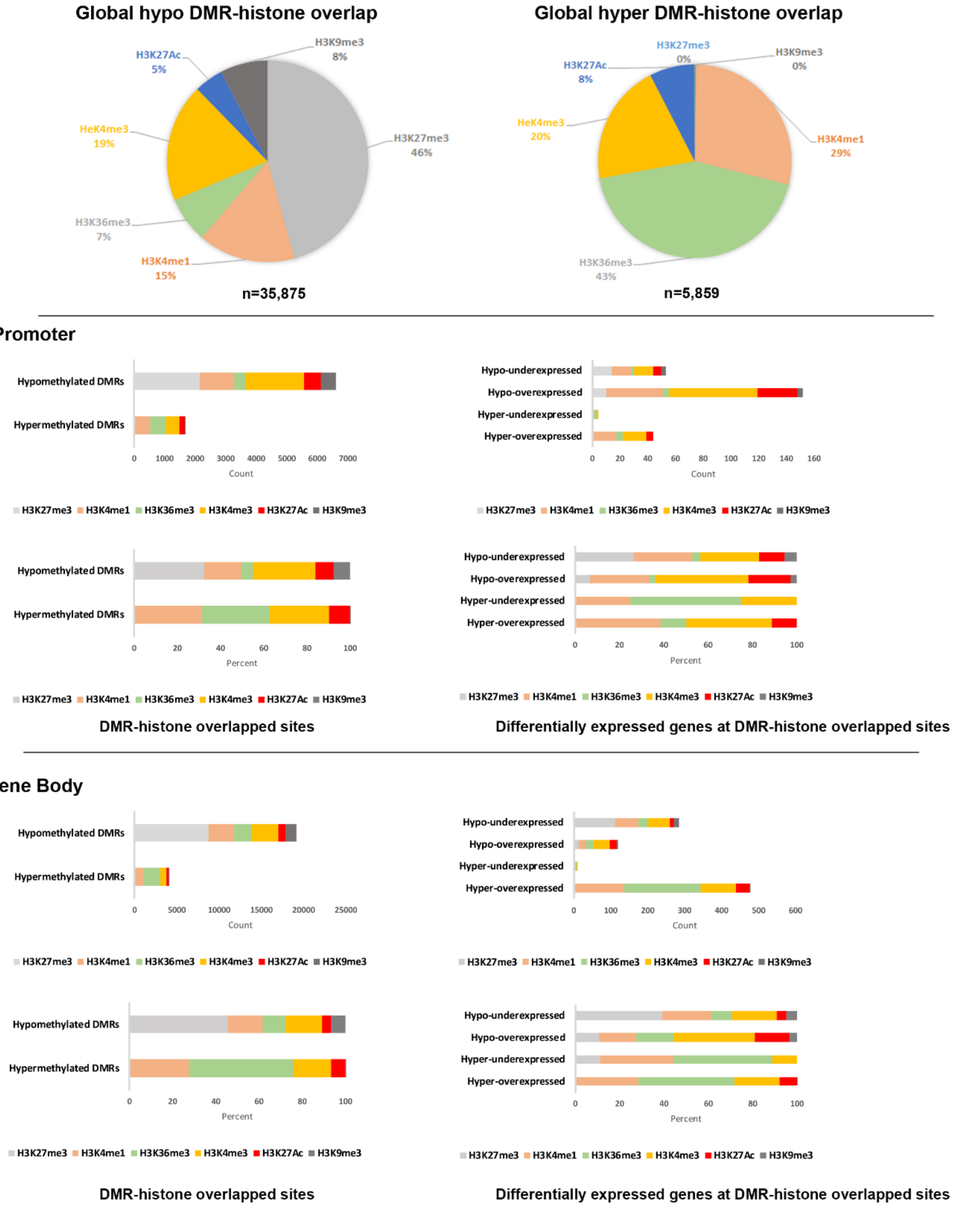

Genomic distribution of DMR and histone overlaps in t(11;14)-U266 cells

Supplementary Figure 7C

Depiction of overlap between the hyper/hypo-methylated DMRs in t(14;16) NDMM patients and histone marks from MM.1S cells. Differentially expressed genes in the t(14;16) subgroup, which spans these DMR-histone overlapped sites are shown at the promoter and gene body as both counts and as a percentage.

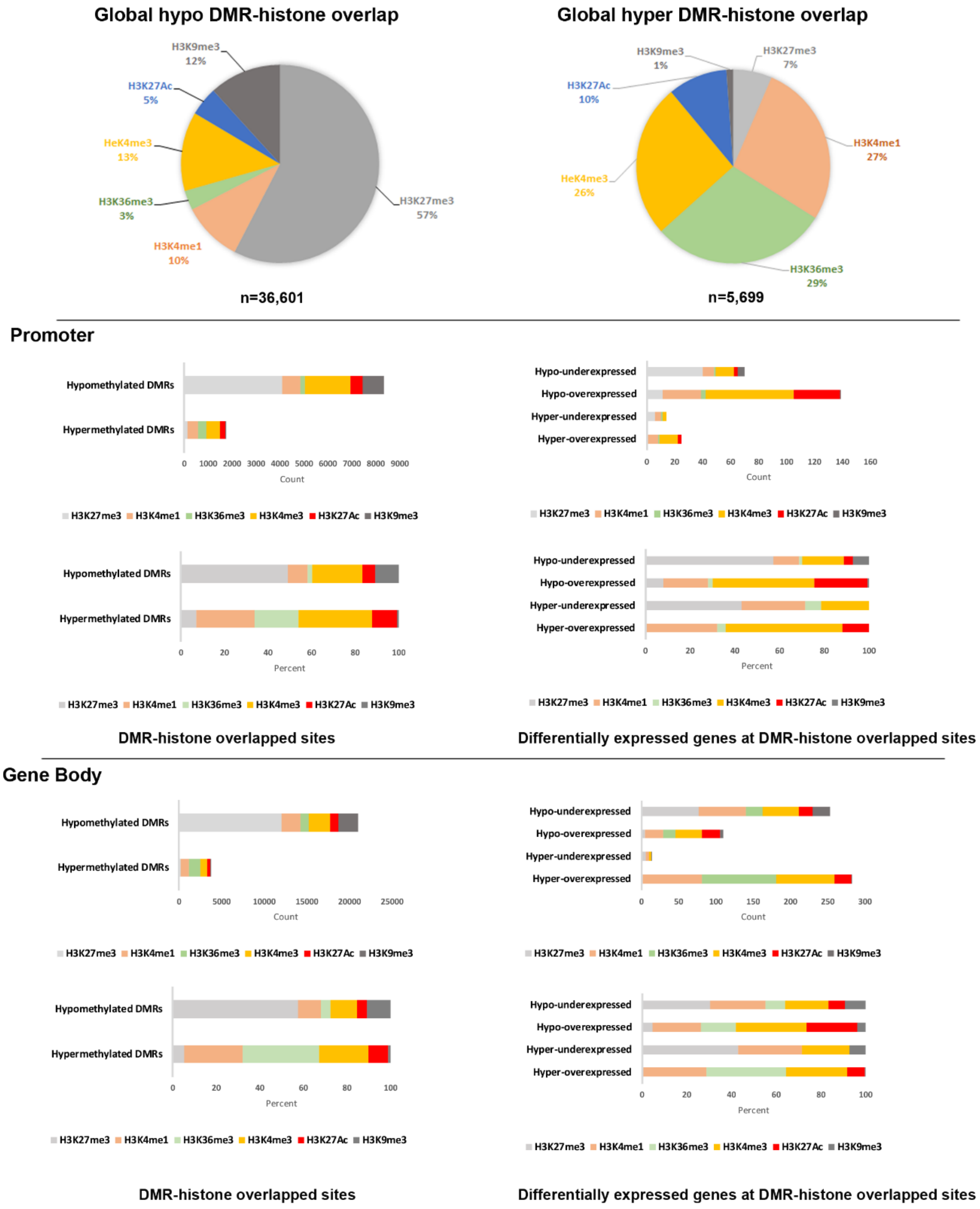

Genomic distribution of DMR and histone overlaps in t(14;16)-MM.1S cells

Supplementary Figure 8

Schematic representation of putative super-enhancer (SE) loops. SE-loops are generally demarcated by CTCF binding sites. SE-loops influence over-expression of an individual gene or a gene cassette, where promoter or upstream promoter regions and gene body could be completely overlapped with the SE elements. The gene expression are also impacted, where SE are not fully overlapped to the gene or the gene-promoter but exist proximally to the gene regulatory elements.

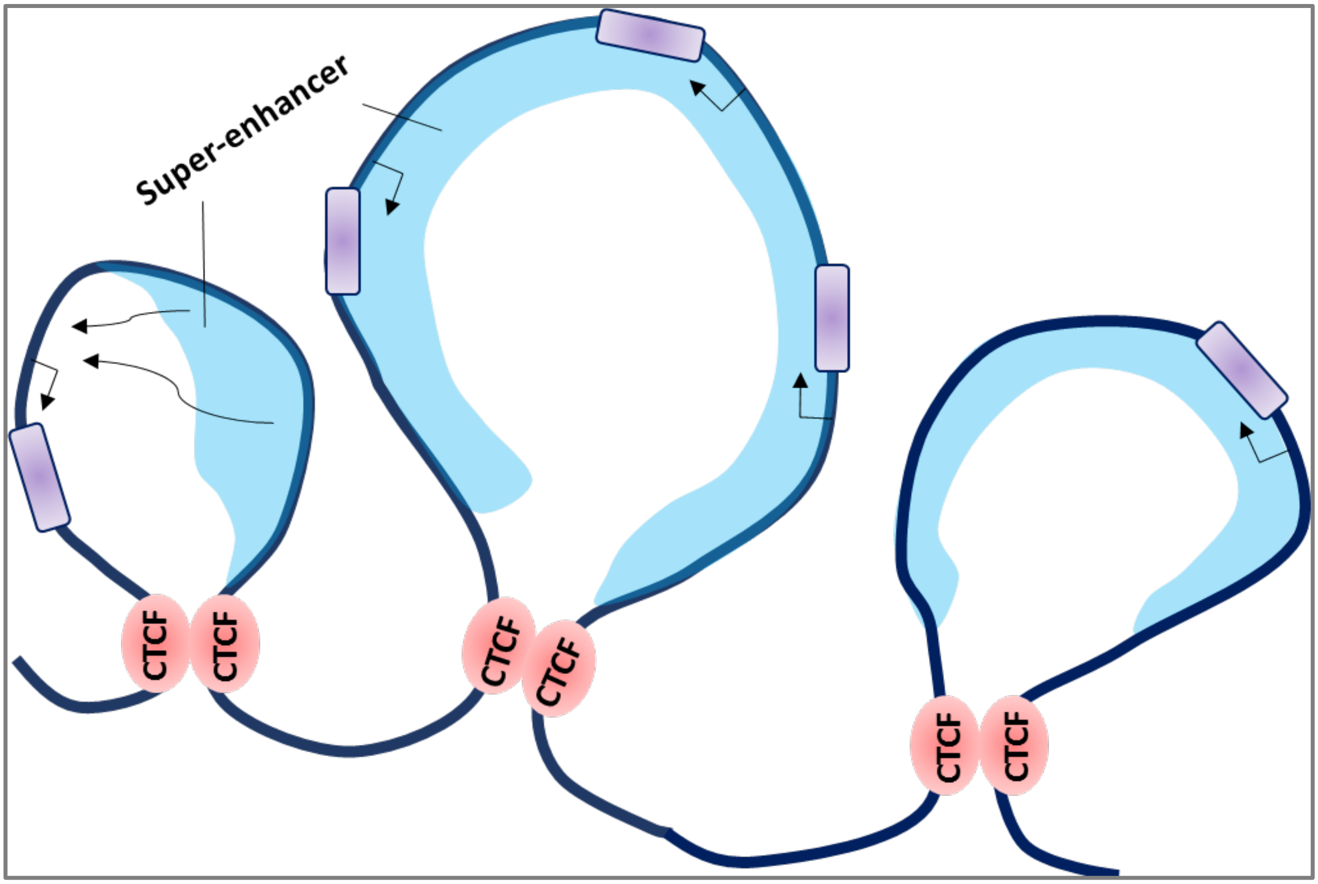

Supplementary Figure 9

The overlap of DMRs (>50 bp) with super-enhancers (SE) (n=677) or CTCF (n=4,791) binding sites was determined at the promoters **(a+d)**, gene bodies **(b+e)** or intergenic regions **(c+f).** Based on average linkage of the methylation values, SE-DMRs were clustered into three major modules [hypermethylated (in blue), hypermethylated (in red) and combination of both, while DMR-CTCFs were clustered into two major modules (hyper- and hypomethylated) across the genomic regions.

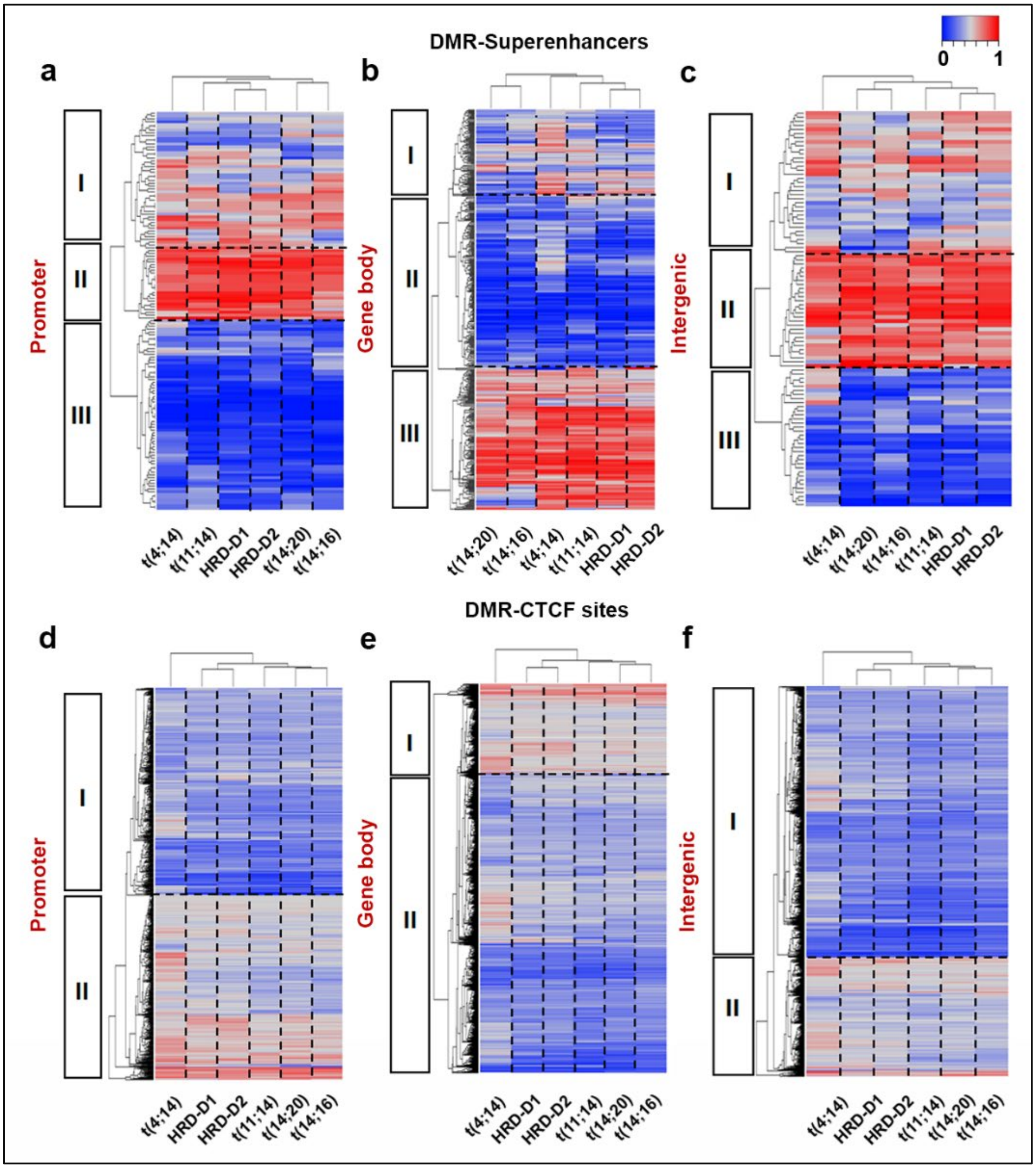

Supplementary Figure 10

MAF overexpression is a signature for the t(14;16) subgroup of MM. We observed an overlap of SE elements at the promoter, upstream enhancer and portion of MAF gene body in MM1.s cells.

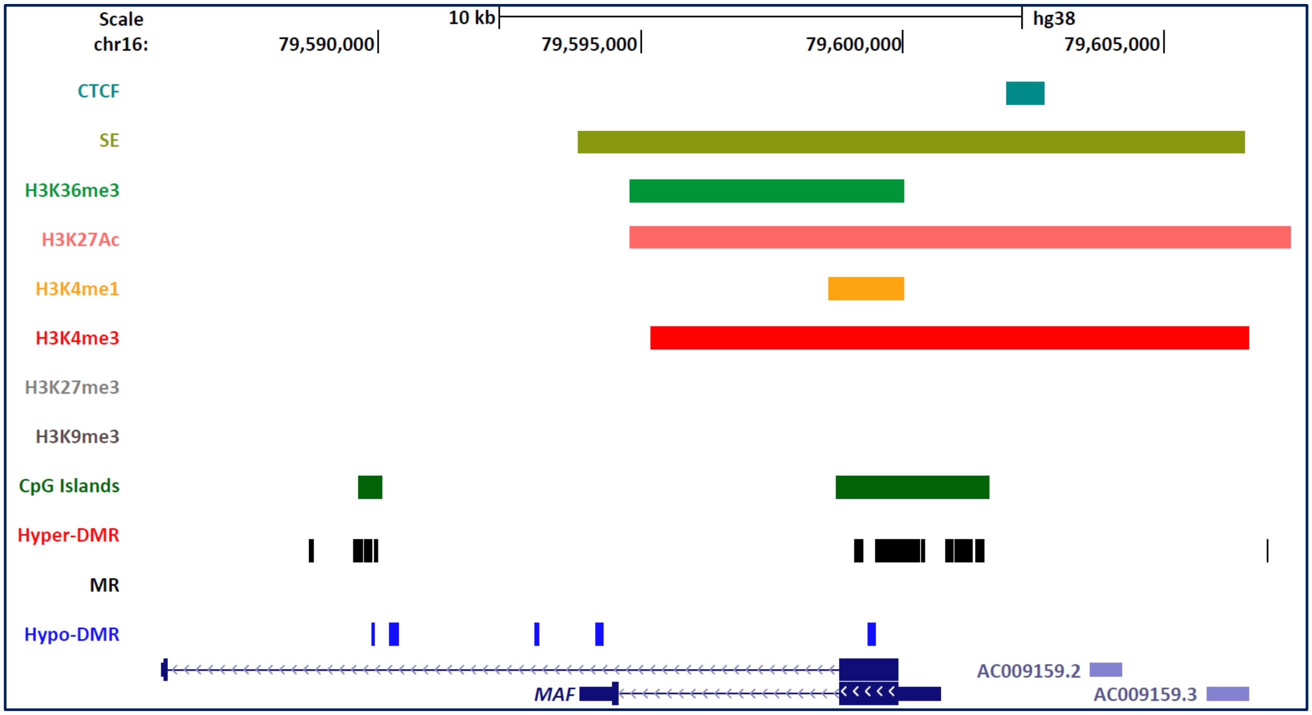

Supplementary Figure 11

Expression of GEMs in NDMM patients were validated using RNA sequencing, based on their correlation (r^2) coefficient. Expression are represented as signal intensity (log2 scale) for GEP and TPM (Transcripts per kb-M). Expression values were color coded based on their intensity values per row.

t(4;14) GEMs

GEP

	t(4;14)	t(11;14)	t(14;16)	t(14;20)	HRD-D1	HRD-D2
TEAD1	5.99	4.23	4.23	4.28	4.44	4.54
C1orf21	6.88	6.78	6.1	6.24	6.11	6.86
CST3	7.64	7.11	6.56	6.53	6.48	6.82
DSG2	8.37	5.98	4.63	5.32	4.94	4.75
JAM3	6.1	5.44	5.38	5.4	5.52	5.46
LARP6	5.9	5.48	5.06	5.04	5.28	5.17
LRP12	5.42	5.38	3.82	3.85	4	5.79
MPPED2	6.06	4.4	3.89	3.83	3.96	4.67
MYRIP	5.97	4.42	4.45	4.38	4.54	5.54
NRIP1	6.9	6.69	6	5.52	4.74	5.12
SYK	5.9	5.68	7.32	7.05	7.32	5.86
TEAD1	5.99	4.23	4.23	4.28	4.44	4.54

RNA seq.

t(4;14)	t(11;14)	t(14;16)	t(14;20)	HRD-D1	HRD-D2	R^2
13.99	0.24	0.28	1.52	21.00	7.58	0.484224
17.96	16.50	7.22	8.67	3.49	34.16	0.836312
112.41	32.36	36.49	44.64	15.41	57.39	0.829439
47.21	9.69	1.12	5.47	2.56	3.59	0.981273
6.29	1.09	0.71	0.90	0.97	1.22	0.988577
2.06	0.72	0.18	0.27	0.46	0.65	0.948859
10.10	5.52	2.40	1.46	3.10	14.41	0.897931
11.72	2.50	1.32	1.73	7.29	2.45	0.76476
19.26	3.22	0.67	0.72	1.57	4.57	0.860656
17.74	11.04	14.19	8.54	5.47	6.70	0.874466
3.81	10.06	39.02	44.58	42.78	27.11	0.876478
13.99	0.24	0.28	1.52	21.00	7.58	0.484224

t(11;14) GEMs

GEP

	t(4;14)	t(11;14)	t(14;16)	t(14;20)	HRD-D1	HRD-D2
PDZRN4	8.20	7.78	4.47	5.24	8.58	6.60
CCND2	9.26	5.50	11.17	10.61	5.78	8.94
NFIB	4.61	4.59	4.47	4.47	4.56	4.51
RFTN1	5.07	4.94	4.99	4.87	5.06	5.09
SORT1	6.37	7.56	6.63	5.87	6.62	7.00

RNA seq.

t(4;14)	t(11;14)	t(14;16)	t(14;20)	HRD-D1	HRD-D2	R^2
38.68	20.39	2.92	4.72	19.94	12.39	0.837631
314.73	1.36	2513.92	2298.98	9.40	356.87	0.836319
1.04	12.31	0.34	0.38	0.76	1.83	0.489436
1.64	1.45	4.76	12.49	2.09	52.24	0.359774
20.40	47.02	31.76	12.59	13.84	32.33	0.887733

maf GEMs

GEP

	t(4;14)	t(11;14)	t(14;16)	t(14;20)	HRD-D1	HRD-D2
AHNAK	7.12	7.37	8.33	8.61	6.67	7.25
ARHGAP6	4.82	5.62	6.72	7.21	4.94	5.75
ARID5A	7.86	8.06	9.90	10.26	7.76	7.22
GULP1	5.36	4.92	7.60	6.42	5.15	5.80
NUAK1	6.19	5.84	10.36	10.60	5.82	5.71
PRR15	7.77	8.56	9.89	10.24	8.28	8.86
SFN	7.68	7.74	8.58	8.75	7.07	7.35
ATP13A2	0.53	0.72	0.80	0.81	0.70	0.68
BASP1	0.58	0.26	0.24	0.22	0.35	0.23
LCP2	0.29	0.23	0.59	0.45	0.30	0.19
MTSS1	0.55	0.51	0.72	0.73	0.45	0.45
RAPH1	0.66	0.76	0.76	0.81	0.77	0.78
SYNGR1	0.64	0.56	0.59	0.50	0.62	0.66

RNA seq.

t(4;14)	t(11;14)	t(14;16)	t(14;20)	HRD-D1	HRD-D2	R^2
312.96	1134.66	3125.87	5942.07	145.31	461.32	0.938491
1.44	3.59	15.08	25.28	1.79	3.20	0.939725
22.01	25.90	119.46	158.69	19.09	27.49	0.963725
2.94	7.11	23.77	43.93	6.76	8.77	0.653473
1.49	0.20	64.22	96.47	0.10	0.22	0.976392
4.71	10.02	41.67	26.19	13.20	25.09	0.84409
0.42	0.78	4.66	1.14	0.22	0.62	0.660334
15.86	18.45	62.78	83.88	12.96	19.02	0.729596
16.74	7.83	0.70	11.55	26.02	10.85	0.510668
2.18	5.11	30.30	20.64	3.66	3.75	0.950568
2.45	7.04	79.20	83.15	1.99	3.49	0.95746
8.19	11.56	27.65	82.30	3.60	13.98	0.576804
3.57	4.34	0.73	0.25	3.69	9.59	0.724522

HRD-D1 GEMs

GEP

	t(4;14)	t(11;14)	t(14;16)	t(14;20)	HRD-D1	HRD-D2
CLPP	9.41	9.63	9.69	9.56	9.92	9.74
COL4A6	5.47	5.01	4.98	4.92	6.34	5.50
COX7C	11.66	11.50	11.69	11.63	12.00	11.83
ERCC1	8.12	8.31	7.86	8.17	8.78	8.53
VILL	7.49	7.55	7.24	7.48	8.15	7.73
COL4A6	5.47	5.01	4.98	4.92	6.34	5.50
COX7C	11.66	11.50	11.69	11.63	12.00	11.83
ILF3	8.57	8.70	8.89	8.97	8.88	8.83
KIT	6.82	7.65	8.13	11.92	9.95	9.89
LSM7	10.82	10.87	11.06	10.87	11.55	11.13

RNA seq.

t(4;14)	t(11;14)	t(14;16)	t(14;20)	HRD-D1	HRD-D2	R^2
14.29868	16.5084	15.81848	14.2813	19.52347	26.83824	0.576322
5.358304	0.294735	1.744734	0.620853	51.42275	12.87693	0.953855
110.6154	108.1012	126.5594	91.5213	164.8576	187.7116	0.795253
21.79558	25.79847	17.2129	20.14154	35.03449	36.43343	0.93457
19.38626	14.23788	14.45051	12.42858	24.07018	24.34207	0.759814
5.358304	0.294735	1.744734	0.620853	51.42275	12.87693	0.953855
110.6154	108.1012	126.5594	91.5213	164.8576	187.7116	0.795253
88.71121	99.96785	102.934	114.3543	121.0079	114.3354	0.822533
3.285468	5.524299	39.16033	112.6273	64.69732	63.66266	0.98215
17.17515	24.51158	23.28796	21.76655	25.73323	30.90846	0.530225

HRD-D2 GEMs

GEP

	t(4;14)	t(11;14)	t(14;16)	t(14;20)	HRD-D1	HRD-D2
NOL4	4.88	4.89	4.5	4.78	7.36	7.67
TNFAIP2	6.35	6.52	6.71	6.68	6.53	7.65

RNA seq.

t(4;14)	t(11;14)	t(14;16)	t(14;20)	HRD-D1	HRD-D2	R^2
2.340548	6.382563	0.171736	2.833667	11.14056	3.436827	0.573567
6.705204	11.49299	12.68538	12.84577	4.544402	13.3675	0.569933
